# RNA/*a*TNA Chimeras: RNAi Effects and Nucleases Resistance of Single and Double Stranded RNAs

**DOI:** 10.3390/molecules191117872

**Published:** 2014-11-04

**Authors:** Adele Alagia, Montserrat Terrazas, Ramon Eritja

**Affiliations:** 1 Institute for Advanced Chemistry of Catalonia (IQAC-CSIC), CIBER-BBN Networking Centre on Bioengineering, Biomaterials and Nanomedicine, Jordi Girona 18–26, Barcelona 08034, Spain; E-Mail: montserrat.terrazas@irbbarcelona.org; 2 Institute for Research in Biomedicine (IRB Barcelona), Baldiri Reixac 10, Barcelona 08028, Spain

**Keywords:** RNAi, siRNA, 3′-overhang chemical modification, single-stranded siRNA, l-threoninol, 3′-exonuclease, 5′-exonuclease, serum resistance, ApoB gene

## Abstract

The RNA interference pathway (RNAi) is a specific and powerful biological process, triggered by small non-coding RNA molecules and involved in gene expression regulation. In this work, we explored the possibility of increasing the biological stability of these RNA molecules by replacing their natural ribose ring with an acyclic l-threoninol backbone. In particular, this modification has been incorporated at certain positions of the oligonucleotide strands and its effects on the biological properties of the siRNA have been evaluated. *In vitro* cellular RNAi assays have demonstrated that the l-threoninol backbone is well tolerated by the RNAi machinery in both double and single-stranded fashion, with activities significantly higher than those evinced by the unmodified RNAs and comparable to the well-known phosphorothioate modification. Additionally, this modification conferred extremely strong resistance to serum and 3′/5′-exonucleases. In view of these results, we applied this modification to the knockdown of a therapeutically relevant human gene such as apolipoprotein B (*ApoB*). Further studies on the activation of the innate immune system showed that l-threoninol-modified RNAs are slightly less stimulatory than unmodified RNAs.

## 1. Introduction

In the late 1970s, it was discovered that long double-stranded oligonucleotides could efficiently control gene expression [1]. Thirty years later, thanks to the elucidation about the mechanism of RNAi pathway [2], it was demonstrated that the same effect could be produced by synthetic 21–23 nt double-stranded RNAs known as short interfering RNAs (siRNAs) [3]. As result of these findings, the post-transcriptional gene silencing disclosed its enormous therapeutic potential and its usefulness for studying gene function. Since the discovery of the RNAi pathway, much effort has been made in order to gather information on its mechanism of silencing. Once inside the cell the siRNA molecule, which is formed by a sense (or passenger) strand and an antisense (or guide) strand with 3′-dinucleotide overhangs, is incorporated into a protein complex called RNA-Induced Silencing Complex (RISC). Then, the loaded siRNA is unwound and only the antisense is held into the RISC, whereas the passenger strand is released. The antisense strand serves as a template for the recognition and cleavage of the target mRNA [4,5]. In theory, by virtue of their unique sequence, siRNA molecules should be able to discriminate between thousands of cellular mRNAs and control any disease-associated genes [6]. However, despite the great attractiveness of siRNAs as potential therapeutic tool, the use of these RNA molecules *in vivo* faces some key hurdles. One of the most important is their susceptibility to the degradation by exo- and endonucleases, which leads to short half-life in serum. Other problems are related to their poor ability to cross cell membranes and their rapid clearance from the bloodstream. Successful RNA-based therapeutics, especially in systemic applications, depends on the improvement of the pharmacological and the nuclease-resistant properties of siRNAs. Hence, tailored design of potent siRNA molecules, that ensure gene silencing at low concentration with nano/pico-molar IC_50_ values and enhanced half-life, are central issues for therapeutic settings. In addition, a pivotal question to answer is whether the observed effects are specific and not due to unwanted “off-target” effects such as the activation of the immune response [7,8]. Looking back over the years, many groups have concentrated their efforts to address these important issues by using chemically modified siRNAs [9]. On the other hand, distinct approaches, like the application of single-stranded antisense siRNAs (ss-siRNAs), could be an attractive way to circumvent the misincorporation of the passenger strand into the RISC avoiding this critical off-target effect [10,11,12,13]. Recently, a new foldamer named *acyclic* Threoninol Nucleic Acid (*a*TNA), bearing d-threoninol (2-amino-1,3-butanediol) as building block tethered to one of the natural nucleobases A, C, G and T has been developed [14]. This new oligomer, although characterized by more flexible scaffold than the natural DNA/RNA, forms a very stable homoduplex in an antiparallel manner and right-handed structure. Furthermore, Murayama and co-workers reported that a fully modified *a*TNA strand cannot hybridize with the complementary DNA/RNA strand [15]. Thus, in view of these interesting structural properties, in this work we functionalized the 3′-overhangs of a siRNA molecule with two l-threoninol thymine units to explore its effects on the biological activities of the siRNAs. In detail, the main goal of this study is to investigate whether the incorporation of the l-threoninol modification in the strand *termini* can increase the resistance of the siRNAs against serum nucleases and specific exo-nucleases without compromising the efficacy, the potency and the duration of their silencing activity *in vitro*. Finally, we also examined the impact of this modification on the induction of the immune response.

## 2. Results and Discussion

### 2.1. Synthesis of the l-threoninol-thymine Building Block

**Figure 1:**
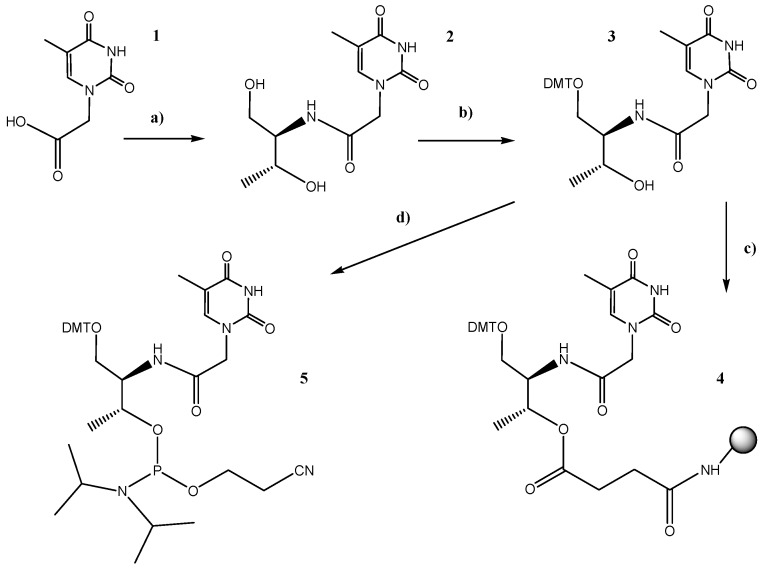
Schematic synthesis of the l-threoninol-thymine, the phoshoramidite and the functionalized solid support.

In order to incorporate two l-threoninol-thymine units at 3′-end of oligonucleotides via solid phase phosphoramidite chemistry, we prepared the succinate derivative needed for the functionalization of controlled pore glass (CPG) solid support (Figure 1). In parallel, with the aim of incorporating the l-threoninol-thymine modification at an internal position of the siRNA, the corresponding phosphoramidite derivative was also synthesized following previously described protocols [14,15] with some small modifications [16]. Reaction between thymine-1-acetic acid (**1**) and *p*-nitrophenol yielded an active ester [17] that reacted with the amino group of l-threoninol. Then, the primary hydroxyl group of the resulting l-threoninol-thymine derivative (**2**) was protected by a 4,4'-dimethoxytrityl (DMT) group to give compound **3**. To enable attachment to the solid support, **3** was first reacted with succinic anhydride. The resulting succinate derivative was then linked to the free amino group of CPG to create the solid support **4** linked to **3**. Moreover, to enable incorporation at an internal position, **3** was phosphitylated by the standard procedure to produce phosphoramidite **5**.

*Reagents and conditions*: (**a**) i: *p*-nitrophenol, DCC, pyridine 0 °C > RT O.N.; ii: l-threoninol, Et_3_N, DMF, RT O.N., 76%; (**b**) DMT-Cl, iPr_2_NEt, pyridine/DMF (4:2) RT 3 h, 76%; (**c**) i: succinic anhydride, DMAP, CH_2_Cl_2_, ii: LCAA-CPG, PPh_3_, DMAP, 2,2'-dithio-bis(5-nitropyridine), CH_2_Cl_2_/ACN; (**d**) 2-cyanoethoxy-*N*,*N'*-diisopropylaminochlorophosphine, iPr_2_NEt, CH_2_Cl_2_, RT, 91%.

### 2.2. RNA Synthesis

To examine whether L-threoninol-modified siRNAs can act as RNAi triggers, two l-threoninol-thymine monomers (**T**^L^) were incorporated at the 3′-*termini* of the antisense (**AS2**) and the sense (**SS2**) RNA strands of the siRNA targeting the *Renilla* luciferase mRNA (Table 1).

**Table 1:** Sequences and mass spectrometry analyses of oligonucleotides. 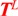: l-threoninol-thymine monomer, 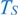: thymidine monomer with phosphorothioate linkage, ***T***: thymidine.

ON	Sequence	MW Calculated	MW Found
AS1	5′-UUUUUCUCCUUCUUCAGAU***TT***	6439	6434
SS1	5′-AUCUGAAGAAGGAGAAAAA***TT***	6829 (+Na)	6829 (+Na)
AS2	5′-UUUUUCUCCUUCUUCAGAU 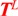 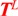	6497	6492
SS2	5′-AUCUGAAGAAGGAGAAAAA 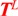 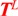	6864	6859
AS3	5′-UCCUUUCUUUCUUUCGAUA***TT***	6439	6433
SS3	5′-UAUCGAAAGAAAGAAAGGA***TT***	6806	6800
SS4	5′- A 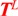 CUGAAGAAGGAGAAAAA***TT***	6833	6827
AS5	5′-UUUCUUGUUCUGAAUGUCC***TT***	6742	6736
SS5	5′-GGACAUUCAGAACAAGAAA***TT***	6518	6512
AS6	5′-UUUCUUGUUCUGAAUGUCC 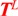 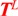	6800	6795
SS6	5′-GGACAUUCAGAACAAGAAA 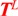 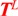	6576	6570
ASP	5′-UUUUUCUCCUUCUUCAGAU 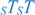	--	--
SSP	5′- AUCUGAAGAAGGAGAAAAA 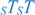	--	--

In order to compare the different potency and efficacy between natural (**AE1**) and different combination of 3′-end modified (**AE2**, **AE3**, **AE4**) siRNAs (Table 2), unmodified antisense and sense strands were also prepared (**AS1** and **SS1**) (Table 1). Moreover, we also assembled unmodified (**APO1**, unmodified sense **SS5** and antisense **AS5** strands) and 3′-modified (**APO6**, modified sense **SS6** and antisense **AS6** strands) siRNA strands targeting the endogenous gene *ApoB* (Table 1 and Table 3). To ensure the specific silencing effects of the siRNAs, scrambled version of antisense and sense strands (**AS3** and **SS3**, respectively) (Table 1) were designed and used as negative control (**SCR**) (Table 2).

**Table 2:** Sequences of unmodified and modified siRNAs targeting the *Renilla* luciferase mRNA and scrambled (SCR) siRNA; Tm (±0.5 °C) and Median Inhibition Concentration (IC_50_) values (mean ± SD). 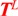: L-threoninol-thymine monomer; 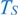: thymidine monomer with phosphorothioate linkage; ***T***: thymidine.

siRNA	ON	Sequence	Tm [°C]	IC_50_ [pM]
AE1	SS1	***TT***AAAAAGAGGAAGAAGUCUA-5′	67.8	9.8 ± 0.2
	AS1	5′-UUUUUCUCCUUCUUCAGAU***TT***		
AE2	SS1	***TT***AAAAAGAGGAAGAAGUCUA-5′	N.D.	6.3 ± 0.5
	AS2	5′-UUUUUCUCCUUCUUCAGAU 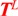 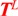		
AE3	SS2	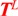 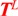 AAAAAGAGGAAGAAGUCUA-5′	N.D.	14.3 ± 0.3
	AS1	5′-UUUUUCUCCUUCUUCAGAU***TT***		
AE4	SS2	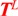 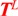 AAAAAGAGGAAGAAGUCUA-5′	67.4	7.2 ± 0.4
	AS2	5′-UUUUUCUCCUUCUUCAGAU 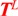 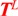		
AES2	SS1	***TT***AAAAAGAGGAAGAAGUCUA-5′	N.D.	6.5 ± 0.2
	ASP	5′- UUUUUCUCCUUCUUCAGAU 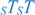		
AES3	SSP	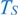 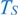 AAAAAGAGGAAGAAGUCUA-5′	N.D.	10.5 ± 0.4
	AS1	5′-UUUUUCUCCUUCUUCAGAU***TT***		
AES4	SSP	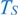 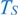 AAAAAGAGGAAGAAGUCUA-5′	67.8	8.3 ± 0.3
	ASP	5′-UUUUUCUCCUUCUUCAGAU 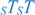		
SCR	SS3	***TT***AGGAAAGAAAGAAAGCUAU-5′	N.D.	Not active
	AS3	5′-UCCUUUCUUUCUUUCGAUA***TT***		

**Table 3:** Sequences of unmodified and **T**^L^-modified siRNAs targeting the *ApoB* mRNA. 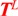: l-threoninol-thymine monomer, ***T***: thymidine.

siRNA	ON	Sequence
APO1	SS5	***TT***AAAGAACAAGACUUACAGG-5′
	AS5	5′-UUUCUUGUUCUGAAUGUCC***TT***
APO6	SS6	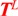 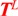 AAAGAACAAGACUUACAGG-5′
	AS6	5′-UUUCUUGUUCUGAAUGUCC 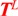 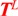

Finally, for 5′-exonuclease studies, one l-threoninol-thymine unit was incorporated at position 2 of the RNA strand (**SS4**) (Table 1). The synthesis of RNAs bearing the modification was realized according to standard solid-phase synthesis protocols.

### 2.3. In Vitro Evaluation of Double-Stranded and Single-Stranded Antisense siRNAs Potency

**Figure 2:**
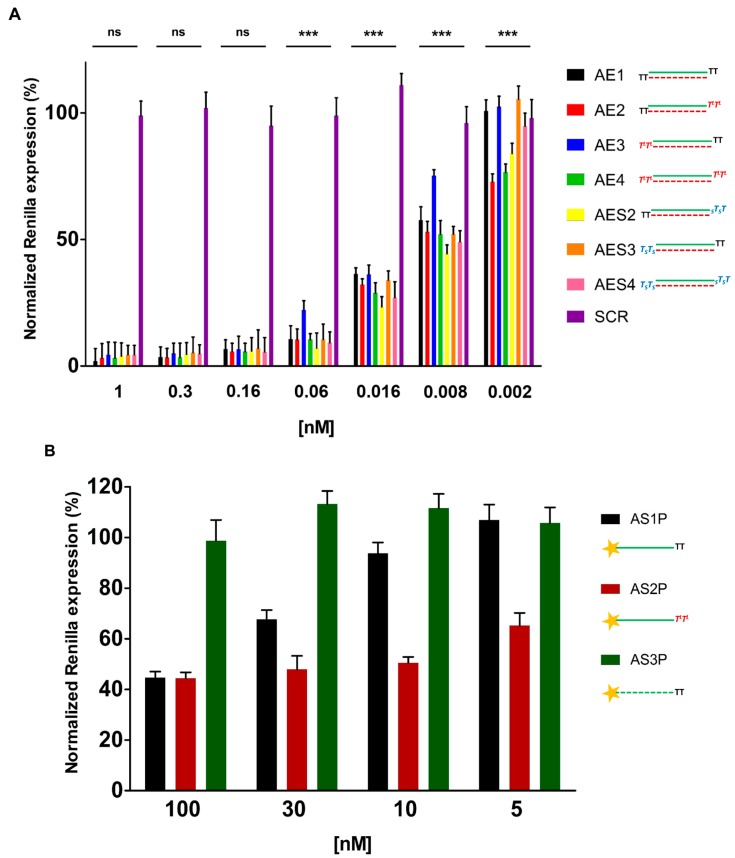
Luciferase assays. (**A**) Dose-response curves of native (**AE1**), **T**^L^ modified (**AE2**, **AE3**, **AE4**) and PS modified (**AES2**, **AES3**, **AES4**) siRNAs. *n* = 3 ± SD; (**B**) Plot of RNAi activity of native (**AS1P**), 3′-end modified (**AS2P**) and scrambled (**AS3P**) single-stranded antisense siRNAs (ss-siRNA) 5′-phosphorylated. For experimental conditions see the Experimental Section. ns = *p* > 0.05; *** = *p* < 0.001. *n* = 3 ± SD.

Initially, in order to determine whether our modified siRNAs are able to suppress gene expression, we carried out RNAi experiments in HeLa cells with unmodified and modified *Renilla* luciferase double-stranded siRNAs (**AE1**, **AE2**, **AE3**, **AE4**, **AES2**, **AES3** and **AES4**) (Table 2). Cells were co-transfected with two vectors carrying *Renilla* and *Firefly* luciferase genes and decreasing concentration of siRNAs. Twenty-four hours after transfection, luminescence was measured. Remarkably, all double-stranded siRNAs used in this study were potent inhibitors of Renilla activity with picomolar IC_50_ values (Figure 2A and Table 2). Very interestingly, the siRNA containing two **T**^L^ units in the 3′-overhang of the antisense strand (**AE2**) displayed gene-silencing (IC_50_ = 6.3 pM) significantly higher than that of the siRNA carrying two natural thymine (**AE1**) (IC_50_ = 9.8 pM) and nearly comparable respect to the siRNA bearing two phosphorothioate linkages at 3′-end of the antisense strand (**AES2**) (IC_50_ = 6.5 pM). For the siRNA containing two **T**^L^ units in the 3′-dinucleotide overhang of the sense strand (**AE3**), the gene-silencing was slightly less efficient than that observed for both unmodified siRNA **AE1** and siRNA modified on the sense strand at 3′-end with two phosphorothioate linkages (**AES3**) (IC_50_ = 10.5 pM). However, despite this loss of activity, siRNA **AE3** retained a significant inhibitory effect (IC_50_ = 14.3 pM). Finally, the siRNAs modified on both overhangs (**AE4** and **AES4**) mainly revealed similar potency (IC_50_ = 7.2 pM and 8.3 pM respectively). Thereafter, we decided to analyze the gene-silencing capability of our modified siRNAs in a single-stranded fashion. Many reports [10,11,12,13] have demonstrated that single-stranded antisense siRNAs (ss-siRNAs) can activate the RNAi pathway. The silencing activity of ss-siRNAs, although less potent than that of their dsRNA counterparts, is strictly dependent on 5′-end phosphorylation. Thus, before transfection, ss-siRNAs (**AS1**; **AS2**; **AS3**) (Table 1) were 5′-phosphorylated by T4 polynucleotide kinase (3′-phosphatase minus) (to give **AS1P**; **AS2P**; **AS3P**). HeLa cells were transfected with the 5′-phosphorylated ss-siRNAs and 24 h post-transfection luminescence was measured. As expected, although retaining consistent silencing ability, all the 5′-phosphorylated ss-siRNAs are weaker effector of RNAi, compared to the double-stranded counterparts. The unmodified ss-siRNA (**AS1P**) and the l-threoninol-modified analogue (**AS2P)** displayed similar *Renilla* inhibitory activity at the highest ss-siRNA concentration (100 nM) (Figure 2B). Very interestingly, the inhibitory activity of the modified ss-siRNA **AS2P** was not significantly affected by decreased concentration of ss-siRNA (30 nM; 10 nM; 5 nM), whereas the native ss-siRNA (**AS1P**) exhibited weaker gene-silencing activity. Remarkably, the modified ss-siRNA (**AS2P**) is about 4-fold more potent than the native one (**AS1P**). HeLa cells transfected with the scrambled ss-siRNA (**AS3P**) showed luminescence levels similar to those of untreated cells. It is noteworthy to mention that at concentrations of 100 nM, 30 nM, 10 nM and 5 nM non-phosphorylated ss-siRNAs (**AS1**; **AS2**; **AS3**) showed no effects on *Renilla* expression (Supplementary Figure S7A).

### 2.4. Over Time Silencing Activity Comparison of siRNAs Targeting Renilla

**Figure 3:**
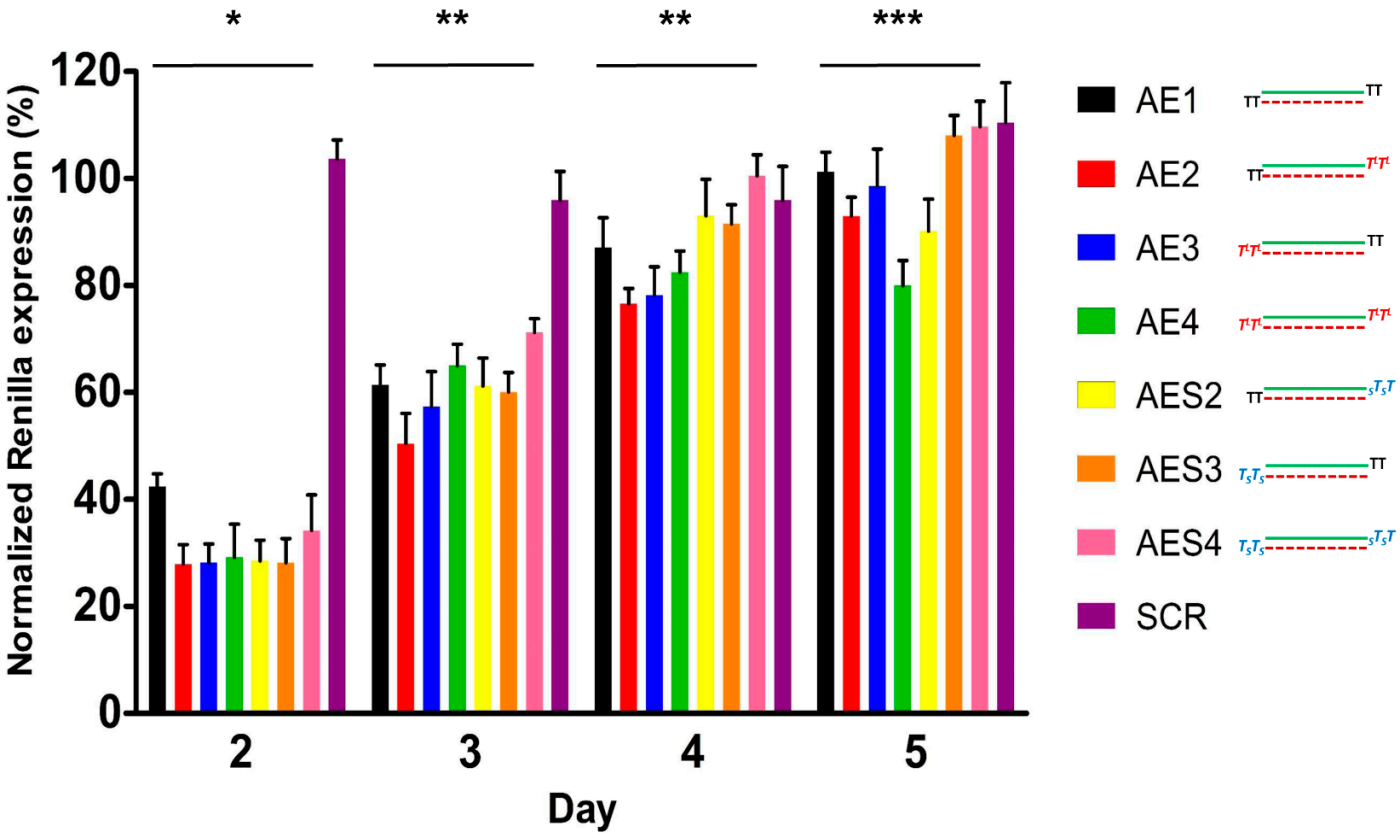
Time course in HeLa H/P. *n* = 3 ± SD. * = *p* < 0.05; ** = *p* < 0.01; *** = *p* < 0.001.

Next, we performed a time course analysis of different siRNA molecules over the time of five days, to evaluate their long term inhibitory properties. HeLa H/P cells stably overexpressing the *Luciferase* and *Renilla* vectors were transfected with 20 nM of siRNAs (**AE1**, **AE2**, **AE3**, **AE4**, **AES2**, **AES3**, **AES4** and **SCR**). Twenty-four hours later, cells were splitted, parallel cultures were maintained without any further treatments and cell pellets were collected at certain time points. Luminescence was assessed as described in the Experimental Section. The gene silencing activities of all the tested siRNAs decreased over time (Figure 3). Starting from day 3, the **AE4** siRNA (containing two l-threoninol units at both 3′-ends) displayed higher activity than siRNAs **AE1** (native) and **AES4** (modified at both 3′-ends with two phosphorothioate linkages). Moreover, at day 5, **AE4** siRNA preserved about 20% of RNAi activity, meaning longer-lasting effects compared to both unmodified **AE1** and phosphorothioate-modified **AES4**. Of note, unlike siRNAs modified on sense strand **AE3** and **AES3**, siRNAs modified on antisense strand **AE2** and **AES2**, still retained slight levels of activity after 5 days of incubation.

### 2.5. l-threoninol Modified siRNA Silencing Depends on Ago2-Mediated Mechanism

The Argonaute 2 protein (Ago2) is the catalytic core of the RISC complex and is responsible for messenger RNA cleavage activity. The perfect base-pairing between siRNA and the targeted mRNA is a prerequisite for Ago2-mediated cleavage [18,19]. As a consequence of the mRNA degradation the protein synthesis is also inhibited. In view to demonstrate that the l-threoninol modified siRNAs act through an Ago2-mediated mechanism, we measured the mRNA levels of the *Renilla* gene after the transfection of siRNAs into wild type Mouse Embryonic Fibroblast (MEF^wt^) and Mouse Embryonic Fibroblast knockout for *Ago2* gene (MEF^Ago2−/−^). In detail, 1 nM and 16 pM of **AE1** and **AE4** siRNAs were transfected and 24 h later the levels of *Renilla* mRNA were measured. As predicted, MEF^Ago2−/−^ transfected with different concentrations of **AE1** and **AE4**, showed no significant changes in *Renilla* expression levels compared to **MOCK** (Supplementary Figure 8SA). In contrast, MEF^wt^ transfected with **AE1** and **AE4** showed a dose dependent silencing of the *Renilla* mRNA (Supplementary Figure S8B). Taken together, these results indicated that the l-threoninol modification is consistent with gene silencing Ago2-dependent.

### 2.6. Effect of l-threoninol Modified siRNA on the HeLa Cell Survival

The proliferation potential of HeLa cells following the transfection of unmodified **AE1** and l-threoninol modified **AE4** was tested by MTT method. 4 siRNA doses (1, 10, 50 and 100 nM) either with the siRNAs complexed with lipofectamine (**AE1L** and **AE4L**) either with siRNAs alone (**AE1** and **AE4**) were examined. As control, untreated cells (**UNT**) and mock transfected cells (**MOCK**) were used. As illustrated in Supplementary Figure S9, MTT assay revealed no significant cytotoxicity due to siRNA transfection in presence or in absence of transfection reagent (**AE1L**, **AE4L**, **AE1** and **AE4** respectively) even at the highest dose applied (100 nM). Hence, the transfection of the native (**AE1**) and the l-threoninol modified siRNA (**AE4**) did not alter the normal proliferation rate of HeLa cells.

### 2.7. Human Serum Nucleases Stability of Chemically Modified siRNAs

The therapeutic application of siRNA depends not only on an efficient gene-silencing activity but also on satisfactory bio-stability. Prior to entering the cell and inducing gene silencing, siRNA molecules must face with extracellular environment such as bloodstream. SiRNAs are highly vulnerable to serum nucleases (endo-, 5′-exo, 3′-exonucleases and RNases), this causes short half-life in serum limiting their application in common therapeutic routes [20]. Chemical modification of siRNA overhangs is a well-accepted approach to enhance its nuclease resistance [21]. Routinely, the stability of siRNAs towards extracellular environment is assayed by the incubation in human blood serum, an excellent mimic of extracellular conditions *in vivo*. Thus, we evaluated the resistance of our modified siRNAs *versus* the action of nucleases by incubation in 90% human serum. 

**Figure 4:**
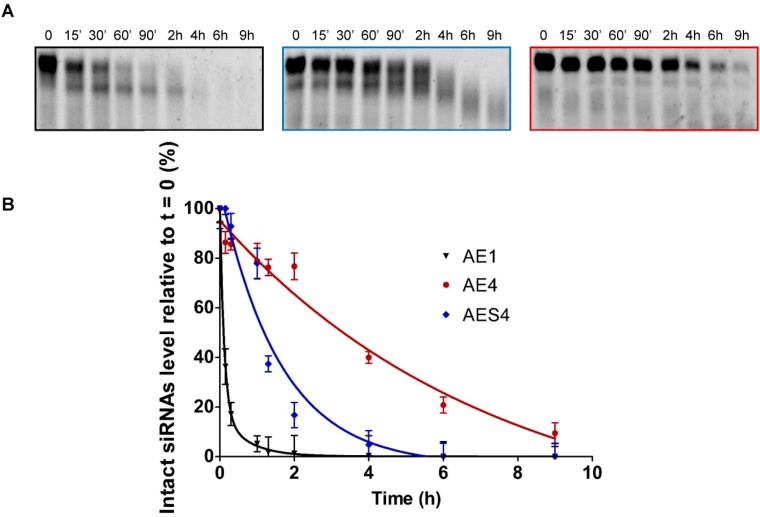
Human serum stability of unmodified and modified siRNAs (**A**) Nucleases stability of native (**AE1**) (left panel), double PS modified (**AES4**) (central panel) and double modified (**AE4**) (right panel) siRNAs; (**B**) Representative degradation curves (**AE1** (black line), **AE4** (red line), **AES4** (blue line)) of human serum assay. Error bars indicate ± SD; *n* = 3. For statistical data analysis and experimental procedures see the Experimental Section.

As expected, unmodified siRNA (**AE1**) (Figure 4A, left panel), was completely degraded after about 60 min of incubation, whereas **AES4** siRNA (Figure 4A, central panel) displayed higher resistance with complete degradation after 4 h. Remarkably, **AE4** (Figure 4A, right panel) showed exceptional nucleases stability, with ~5% of the original siRNA remaining intact after 8 h. The plotted percentage of intact siRNAs over the incubation time permitted us to assess the half-life in about 30 min for **AE1**, 1.3 h for **AES4** and 5.4 h for **AE4** (Figure 4B). Indeed, **AE4** siRNA is 10-fold more resistant respect to **AE1** and 4.5-fold more resistant than **AES4**. Finally, we checked the integrity of **AE1** siRNA (Supplementary Figure 10S) to exclude degradation serum-independent.

### 2.8. 3′-/5′-Exonuclease Resistance Studies of Modified ssRNAs

**Figure 5:**
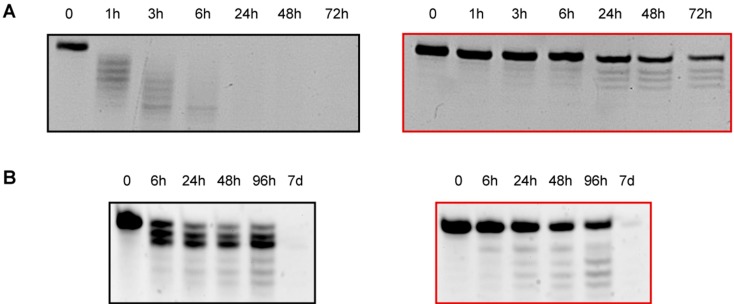
Stability of unmodified and modified ssRNAs against 3′- and 5′- exonucleases (**A**) SVPD degradation of native (**AS1**) (left panel) and 3′-end modified (**AS2**) (right panel) single stranded RNAs; (**B**) BSP degradation of native (**SS1**) (left panel) and position 2 modified (**SS4**) (right panel) single stranded RNAs. For experimental conditions see Material and Methods.

The degradation of siRNAs in serum is mainly due to the action of endoribonucleases belonging to RNAses-A family. However, a slower hydrolytic process, resulting from the attack of exonucleases, participates to the shortening of siRNA starting from their *termini* [22,23]. In order to investigate the effect of our modifications on these exonuclease cleavage processes, we studied the stability of single-stranded **T**^L^-modified RNAs against two well-studied phosphodiesterases that have catalytic activities similar to those of pyrophosphatases/phosphodiesterases present in serum [24]: Snake Venom Phosphodiesterase I (SVPD) and Bovine Spleen Phosphodiesterase II (BSP). SVPD and BSP hydrolyze single stranded RNAs starting from the 3′-end and the 5′-end, respectively. Interestingly, incubation of native (**AS1**) and modified (**AS2**) ssRNAs with SVPD revealed that the 60% of **T**^L^-modified ssRNA (**AS2**) remained intact after 72 h (Figure 5A, right panel).

In contrast, unmodified ssRNA (**AS1**) was completely degraded within 1 h (Figure 5A, left panel) confirming that the presence of two **T**^L^ at positions 20 and 21 strongly enhances the resistance to 3′-exonuclease digestion. On the other hand, quantification of the bands proved that in the case of unmodified ssRNA (**SS1**) (Figure 5B, left panel), only the 22% of the original RNA remains intact after 96 h of incubation with BSP, in contrast to the 55% of **SS4** (Figure 5B, right panel). Moreover, the degradation pattern of **SS4**, containing a **T**^L^ unit at position 2, revealed the absence of the band corresponding to 20-mer degraded ssRNA (Figure 5B, right panel). This phenomenon could be explained by the fact that the presence of the modification at position 2 might prevent the cleavage of the first phosphodiester linkage. Hence, the **T**^L^ modification also confers greater resistance to degradation of 5′-exonucleases such as BSP.

### 2.9. Keeping the Silencing: Evaluation of Long-Term RNAi Activity on ApoB Gene

It has been reported that gene-silencing can be effective up to one month [25], the reason of prolonged silencing could reside either in the protective action of the RISC protein complex as well as in the accumulation into specific intracellular *foci* such as the P-bodies or in a slow cellular proliferation rate [8,26,27]. Thus, gene silencing mediated by siRNA is basically due to the cellular doubling-time and consequent serial dilution of the intracellular siRNA pool. Indeed the lengthening of half-life of modified siRNAs does not imply more durable silencing effects [25,28,29]. On the other hand, it has been reported [30], that in an intracellular environment, double-stranded siRNAs (ds-siRNAs) are more stable than the single-stranded siRNA (ss-siRNA) counterparts. Such different sensitiveness to nuclease degradation reflects, in some extent, distinct silencing activity. Hence, indirect evidence on the authentic stability of ss-siRNAs can be extrapolated comparing their gene-silencing ability. Firstly, we compared the long-lasting RNAi activity of native (**APO1**) and modified (**APO6**) ds-siRNAs against the endogenous *ApoB* gene [31].

**Figure 6:**
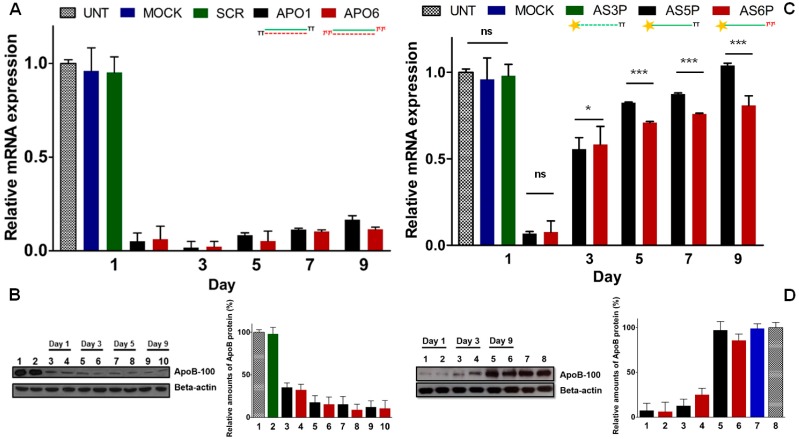
Knockdown of the endogenous *ApoB* gene in HepG2 cells. (**A**) *ApoB* mRNA reduction with 60 nM of unmodified **APO1** and modified **APO6** ds-siRNAs; (**B**) Western blot and quantification plot of ApoB protein expression after siRNAs transfection. Lane 1: Untransfected; lane 2: Scrambled; lanes 3, 5, 7, 9: **APO1** siRNAs; lanes 4, 6, 8, 10: **APO6** siRNAs; (**C**) *APOB* mRNA levels with 60 nM of 5′-phosphorylated unmodified (**AS5P**) and modified (**AS6P**) single-stranded siRNAs; (**D**) Western blot and quantification plot of ApoB protein levels after treatment with ss-siRNAs (60 nM). Lanes 1, 3, 5: **AS5P** ss-siRNAs; lanes 2, 4, 6: **AS6P** ss-siRNAs; lane 7: Mock transfection; lane 8: Untransfected. *n* = 3 ± SD. ns = *p* > 0.05; * = *p* < 0.05; *** = *p* < 0.001. For quantification of ApoB protein levels, untransfected cells were set at 100%. Error bars indicate ± SD, *n* = 2. For experimental conditions see the Experimental Section.

To evaluate the effectiveness of our siRNAs over the time, we reverse-transfected HepG2 cells, which naturally express high level of the *ApoB* gene, [32] with 60nM of **APO1** and **APO6**. The *ApoB* mRNA and protein levels were analyzed at certain times by RT-qPCR and western blotting. As shown in Figure 6A, at 24 h *ApoB* mRNA levels were strongly down-regulated by both unmodified (**APO1**) and modified (**APO6**) siRNAs. Of note, the levels of *ApoB* mRNA were even lower at 72 h after transfection, due to the extremely long half-life of *ApoB* mRNA, estimated of about 16 h. According to previous studies, underlying the correlation between prolonged silencing activity and low cell division rates, we noted that, in HepG2 cells which divide approximately each 48 h [33], the silencing persisted more than 9 days (Figure 6A). Moreover the magnitude of knockdown is not dose-dependent, remaining nearly the same for both **APO1** and **APO6**. As expected, no relevant down-regulation in cells treated with transfection reagent only (**MOCK**) and control siRNA (**SCR**) was observed, denoting an absence of artifacts derived from sequence independent mechanisms. Western blot analyses (Figure 6B) confirmed the strong reduction of ApoB-100 protein levels, during the course of 9 days after transfection of **APO1** and **APO6** siRNAs. No appreciable reduction of ApoB protein levels in cells treated with control siRNA (**SCR**) was noted. The time course analysis revealed no substantial difference between the performance of the native siRNA **APO1** and the modified analogue **APO6**. Thus, to demonstrate that the l-threoninol-thymine modification actively contributes to more durable silencing effects, we reverse-transfected HepG2 cells with 60 nM of 5′-phosphorylated single-stranded antisense siRNAs (ss-siRNAs) (**AS5P** and **AS6P**). The *ApoB* mRNA and protein levels were quantified at certain time points by RT-qPCR and western blotting, respectively. At day 1, the knockdown of the *ApoB* mRNA reached more than 90%, but along the days, the silencing effects of ss-siRNAs disappeared more rapidly than the ds-siRNA counterparts (Figure 6C). Interestingly, at day 9 the modified ss-siRNA **AS6P** still preserved about 20% of *ApoB* knockdown, whereas the unmodified ss-siRNA **AS5P** was no longer effective (Figure 6C). Similar knockdown pattern was observed looking at the protein levels (Figure 6D). No appreciable silencing was found when HepG2 cells were reverse-transfected with 60 nM of no 5′-phosphorylated ss-siRNAs (**AS5** and **AS6**) (Supplementary Figure S7B).

### 2.10. siRNA-Mediated Innate Immune System Activation

**Figure 7:**
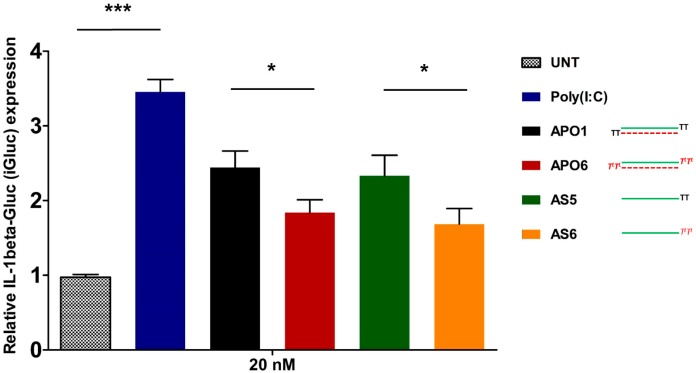
Assessment of IL-1beta-Gluc (iGluc) production siRNA-mediated after transfection of unmodified (**APO1** and **AS5**) and modified (**APO6** and **AS6**) double and single-stranded siRNAs. Transfection of 50 ng/mL of Poly (I:C) was designated as **Poly (I:C)**. *n* = 3 ± SD. * = *p* < 0.05; *** = *p* < 0.001. For experimental conditions see the Experimental Section.

Despite the benefits of the induction of the interferon response in some clinical application, the activation of the innate immune system response by siRNA is an important aspect to avoid for the safe therapeutic use of them [34,35]. The human immune system has evolved to recognize exogenous ds-RNAs as a hallmark of viral infection, thus the intracellular presence of siRNAs could be taken for a dangerous signal of viral attack, inducing a potent and deleterious production of pro-inflammatory and antiviral cytokines (IL-18, IL-1beta, and IFN-beta) and the up-regulation of hundreds of interferon-stimulated genes (ISGs). Several reports have demonstrated that the stimulation of the immune system by siRNA molecules depends on different factors: the chemical structure and the length of siRNA and its specific nucleotide sequence, the relative concentration of siRNA at the time of transfection and the cell type involved [36,37,38]. The classical experimental approach for the evaluation of Type I interferon response, requires the monitoring of certain cytokine production (IFN-beta, IL-6, TNF-alpha), but its assessment can be underestimated because of the short half-lives of these cytokines. An alternative approach for the evaluation of siRNA-induced immune stimulation consists in the screening of the mRNA levels of several Interferon Stimulated Genes (ISGs) by RT-qPCR [39]. It has been reported that the 2′-hydroxyl group of the uridine ribose triggers the immune response [40]. Based on these findings, we explored the possibility of decreasing immunostimulation by protecting the 3′-ends of the RNA strands with l-threoninol-thymine moieties. With the aim of evaluating the overall Type I interferon response caused by our siRNAs, we monitored some of the most notable ISGs (PKR, IFITM1, MX1, OAS1 and ISG56). As shown in Supplementary Figure 11SA, no significant up-regulation of the considered genes was observed, after the transfection of HepG2 cells with modified (**APO6**) and unmodified (**APO1**) siRNAs at different concentrations (40 nM; 60 nM; 100 nM), compared to untreated control cells, whereas cells treated with poly I:C (50 ng/mL) produced a consistent up-regulation of all genes considered. Moreover, similar results have been achieved by the transfection of THP-1 cells [41] with both modified (**APO6**) and unmodified (**APO1**) siRNA at concentration of 60 nM (Supplementary Figure 11SB). As alternative experimental approach to assess the activation of the Interferon response, we decided to monitor the induction of the pro-inflammatory cytokine IL-1beta. To this aim we used THP-1 C1 cells, [42] which are a useful tool to follow the production of IL-1beta [43]. In fact the presence of the fusion protein pro-IL-1beta-GLuc facilitates the measurement of the immune response stimulation, resulting in rapid and reliable changes of the luciferase luminescence. Thus, we transfected THP-1 C1 cells with both double and single stranded siRNAs, as it was demonstrated that double-stranded siRNAs could fail to induce a clear inflammatory response as their single-stranded siRNA counterparts. Oppositely to ISGs screening, Figure 7 illustrated a consistent over-expression of the IL-1beta respect to untransfected THP-1 C1 cells (**UNT**). Poly (I:C) transfected cells were used as positive control. In detail, the induction of IL-1beta production was stronger in the case of unmodified ds-siRNA (**APO1**) respect to the modified one (**APO6**). Similar results were obtained after the transfection of ss-siRNAs, the unmodified **AS5** yielded to a higher production of IL-1beta respect to the modified one (**AS6**). Despite previous reports, the comparison of IL-1beta production siRNAs-mediated in double and single-stranded fashion are quite equivalent.

## 3. Experimental Section

### 3.1. Abbreviations and Acronyms

ACN: acetonitrile, Ac: acetyl, Ac_2_O: acetic anhydride, AcOH: acetic acid, ApoB: apolipoprotein B; *a*TNA: acyclic threoninol nucleic acids, Bz: benzoyl, DMAP: *N*,*N*-dimethylaminopyridine, dmf: dimethylformamidino, DMF: *N*,*N*-dimethylformamide, DMT: dimethoxytrityl, Et_3_N: triethylamine, ES: electrospray, GAPDH: glyceraldehyde 3′-phosphate dehydrogenase, HEPES: 4-[(2-hydroxyethyl)-1-piperazin-1-ylethane]sulfonic acid, HRMS: high resolution mass spectrometry, IFITM-1: interferon-induced transmembrane protein 1, ISG56: interferon-stimulated gene 56, iPr_2_NEt: ethyldisopropylamine, ISGs: interferon stimulated genes, KAcO: potassium acetate, LCAA-CPG: long amino alkyl controlled pore glass, LDL: low-density lipoprotein, MeOH: methanol, NMR: nuclear magnetic resonance, MX1: interferon induced GTP-binding protein (Myxovirus resistance), OAS-1: 2′,5′-oligoadenylate synthetase 1, PPh_3_: triphenylphosphine, PKR: protein kinase R, RISC: RNA-induced silencing complex, RP-HPLC: reversed phase high performance liquid chromatography, RT-qPCR: reversed transcription quantitative polymerase chain reaction, siRNA: short interfering RNA, SCR: scrambled sequence, TBDMS: *tert*-butyldimethylsilyl, TBST: Tris buffered saline and Tween 20 solution, TEAA: triethylammonium acetate, THAP: 2,4,6-trihydroxyacetophenone, **T**^L^: l-threoninol-thymine monomer, TLC: thin-layer chromatography, Tris: 2-amino-2-hydroxymethylpropane-1,3-diol, VLDL: very-low-density lipoprotein.

### 3.2. General Experimental Methods 

All reagents were purchased from Sigma-Aldrich (Tres cantos, Madrid, Spain) or Fluka (Sigma-Aldrich Química S.A., Tres cantos, Madrid, Spain) and used without further purification. Anhydrous solvents and deuterated solvents (CDCl_3_ and DMSO-*d*_6_) were obtained from Sigma-Aldrich or Fluka and used as supplied. All standard phosphoramidites and reagents for oligonucleotide synthesis were purchased from Applied Biosystem (Foster City, CA, USA) or Link Technologies (Glasgow, Scotland, UK) and used as received. All chemical reactions were carried out under argon atmosphere in oven-dried glassware. Thin-layer chromatography was carried out on aluminum-backed Silica Gel 60 F_254_ plates. Flash column chromatography was performed on silica gel SDS 0.063–0.2 mm/70–230 mesh. ^1^H- ^31^P- and ^13^C-NMR spectra were recorded at 25 °C on a Varian Mercury 400 MHz spectrometer. Chemical shifts are reported in parts per million (ppm); J values are given in hertz (Hz). All spectra were internally referenced to the appropriate residual undeuterated solvent. RP-HPLC purifications were performed using a Nucleosil 120–10 C18 column (250 × 4 mm). UV analyses and melting curves were performed using a Jasco V-650 (Easton, MD, USA) instrument equipped with a thermoregulated cell holder. HMRS spectra were performed on a LC/MSD-TOF (Agilent Technologies, Santa Clara, CA, USA) mass spectrometer. MALDI-TOF spectra were recorded on a Perspective Voyager DETMRP mass spectrometer. The matrix used contained 2,4,6-trihydroxyacetophenone (THAP, 10 mg/mL in CH_3_CN/water 1:1) and ammonium citrate (50 mg/mL in water).

### 3.3. Synthesis of Building Blocks

#### 3.3.1. l-threoninol-thymine

(Step I): To a solution of *p*-nitrophenol (424 mg, 3.05 mmol) and thymine-1-acetic acid (**1**, 468 mg, 2.54 mmol) in pyridine (15 mL), 1,3-dicyclohexylcarbodiimide (629 mg, 3.05 mmol) was added. The temperature was kept at 0 °C for 1 h and then, the mixture was allowed to react at room temperature overnight. The precipitate was filtered and the solvent was evaporated *in vacuo*. Residual pyridine was removed by co-evaporation with toluene followed by ACN. (Step II): The active ester was added to a solution of l-threoninol (295 mg, 2.8 mmol) and Et_3_N (780 μL, 5.6 mmol) in DMF (20 mL) and was stirred for 5 h at room temperature. The solution was evaporated to dryness and residual DMF was removed by co-evaporation with toluene followed by ACN. The residue was purified by silica gel chromatography and the pure product was eluted with CH_2_Cl_2_/MeOH 90:10 as a white solid (591 mg, 78% yield).

^1^H-NMR [DMSO-*d*_6_, 400 MHz] δ 11.20 (bs, 1H, CONHCO), 7.72 (d, *J* = 8.8 Hz, 1H, CHNHCO), 7.39 (m, 1H, H_3_CC(CHNHC)), 4.58–4.55 (m, 2H, HOCH_2_), 4.34 (d, *J* = 16.4 Hz, 1H, (CO)CHAHBNCO), 4.27 (d, *J* = 16.4 Hz (CO)CHAHBNCO), 3.86 (m, 1H, CH_3_CHOH), 3.60 (m, 1H, CH_2_CHNH), 3.44 (m, 1H, OH), 1.73 (d, *J* = 0.8 Hz, 3H, CH_3_C(CO)NH, 0.98 (d, *J* = 6.4 Hz, 3H, CH_3_CHOH). HRMS (ES^+^) C_11_H_17_N_3_O_5_ calculated: 272.2803; found: [M+H]^+^ 272.1246.

#### 3.3.2. DMT-Protected l-threoninol-thymine

Compound **2** (167 mg, 0.61 mmol) was dried by co-evaporation with anhydrous pyridine and then dissolved in anhydrous pyridine (6.6 mL). Then, a solution of iPr_2_NEt (160 μL, 0.92 mmol) and 4,4′-dimethoxytrityl chloride (251 mg, 0.74 mmol) in DMF (3.3 mL) was added dropwise. The reaction mixture was stirred for 30 min on an ice bath and then allowed to proceed at room temperature. After 4 h, the reaction was judged as complete by TLC (CH_2_Cl_2_/MeOH 95:5) and was quenched by addiction of 5% NaH_2_PO_4_ and extracted with CH_2_Cl_2_. The organic layer was dried with MgSO_4_, filtered and concentrated *in vacuo*. The residue that was obtained was purified by silica gel chromatography. The column was packed with 98:2 CH_2_Cl_2_/MeOH and the desired product was eluted with 95:5 CH_2_Cl_2_/MeOH. After the removal of the solvent was eliminated under reduced pressure, the desired pure compound (**3**) was obtained as a white solid (269 mg, 76% yield). ^1^H-NMR [CDCl_3_, 400 MHz] δ 7.38–7.15 (m, 10H, DMT aromatic and CHNHCO), 7.00 (m, 1H, H_3_CC(CHNHC)), 6.80 (d, *J* = 8.0 Hz, 4H, DMT aromatic), 4.44 (d, *J* = 15.6 Hz, 1H, (CO)CHAHBNCO), 4.21 (d, *J* = 15.6 Hz, 1H, (CO)CHAHBNCO), 4.08 (m,1H, CH_3_CHOH) 3.97 (m, 1H, CH_2_CHNH), 3.75 (s, 6H, OCH_3_), 3.31 (dd, *J* = 14.8 Hz, *J* = 5.2, 1H, DMTOCH_2_), 3.19 (dd, *J* = 14.8 Hz, *J* = 5.2, 1H, DMTOCH_2_), 1.82 (s,3H, CH_3_C(CO)NH), 1.08 (d, *J* = 6.4 Hz, 3H, CH_3_CHOH). ^13^C-NMR [CDCl_3_, 75 MHz] δ 168.0, 165.4, 159.5, 152.3, 145.3, 141.8, 136.4, 136.3, 130.7, 128.7, 127.7, 113.9, 111.5, 87.0, 68.9, 64.5, 55.5, 54.5, 53.7, 50.4, 31.1, 20.0, 12.3. HRMS (ES^+^) C_32_H_35_N_3_O_7_ calculated: 574.2554; found: [M+H]^+^ 573.6521.

#### 3.3.3. Solid Support Functionalization

In order to conjugate compound **3** to a long-chain alkylamine-controlled pore glass support (LCAA-CPG), the standard methodology via hemisuccinate derivative was used. Step I): Compound **3** (32 mg, 0.06 mmol) was dried twice by co-evaporation with anhydrous ACN under reduced pressure and was dissolved in anhydrous CH_2_Cl_2_ (4 mL). Succinic anhydride (12 mg, 0.13 mmol) and 4-dimethylaminopyridine (DMAP) (16 mg, 0.13 mmol) were added. After 4 h at room temperature starting material was completely converted to the corresponding monosuccinate derivative (as judged by TLC). After treatment with 5% of NaH_2_PO_4_, the product was extracted with CH_2_Cl_2_ and the organic layer was dried with MgSO_4_, filtered and concentrated. The monosuccinate derivative was used for next step without any further purification. Step II): 2,2′-dithio-bis(5-nitropyridine) (9.5 mg, 0.03 mmol), dissolved in an CH_2_Cl_2_/ACN mixture (3:1, 150 µL) was mixed with a solution of the monosuccinate derivative (20 mg, 0.03 mmol) and DMAP (3.7 mg, 0.03 mmol) in ACN (500 µL). The resulting solution was added at room temperature to a solution of triphenylphosphine (PPh_3_) (8.1 mg, 0.03 mmol) in ACN (100 µL). The mixture was briefly vortexed and then added into a vial containing CPG (CPG Inc., Lincoln Park, NJ, USA) (160 mg, 0.03 mmol) and allowed to react for 3 h at room temperature. The functionalized support was placed onto a sintered glass funnel and washed with ACN (2 × 5 mL) and CH_2_Cl_2_ (2 × 5 mL) and dried under high vacuum. Finally, the derivatized support (**4**) was treated with 500 μL of Ac_2_O/DMF 1:1 to cap free amino groups. The functionalization of the resin was determined by DMT quantification resulting from acid-catalyzed detritylation at 498nm using an UV-visible spectrophotometer (f = 25.6 µmol/g).

#### 3.3.4. Synthesis of the Phosphoramidite Derivative

Compound **3** (250 mg, 0.43 mmol) was dried twice by co-evaporation with anhydrous pyridine under reduced pressure and was dissolved in anhydrous CH_2_Cl_2_ (5 mL). After addition of *N*,*N*-diisopropylethylamine (iPr_2_Net, 330 µL, 1.9 mmol) the reaction mixture was cooled on ice and 2-cyanoethoxy-*N*,*N*′-diisopropylaminochlorophosphine (210 µL, 0.95 mmol) was added dropwise. After stirring for 1 h and 30 min at room temperature, starting material was completely converted to the phosphoramidite derivative as evidenced by TLC. The reaction mixture was washed with 5% NaHCO_3_and extracted with CH_2_Cl_2_. The organic layer was dried with MgSO_4_, filtered and evaporated to dryness. The residue that was obtained was purified by silica gel chromatography. The column was packed using 1:1 ethyl acetate/hexane + 5% triethylamine and eluted with 1:1 ethyl acetate/hexane, to give compound (**5**), as white foam (305 mg, 91% yield). ^1^H-NMR [CDCl_3_, 400 MHz] δ 7.39–7.16 (m, 10H, DMT aromatic and CHNHCO), 7.01 (m, 1H, H_3_CC(CHNHC)), 6.80 (d, *J* = 8.0 Hz, 4H, DMT aromatic), 6.32 (d, 1H, *J* = 8.0 Hz, CH_3_CHOP), 4.38–4.24 (m, 3H, (CO)CHAHBNCO, (CO)CHAHBNCO, CH_3_CHOH), 4.14 (m, 1H, CH_2_CHNH), 3.76 (s, 6H, OCH_3_), 3.50–3.40 (m, 4H, OCH_2_CH_2_CN, CH_3_CHNP), 3.17–3.15 (m, 2H, DMTOCH_2_), 1.82 (s, 3H, CH_3_C(CO)NH), 1.08 (d, *J* = 6.4 Hz, 3H, CH_3_CHOH). ^13^C-NMR [CDCl_3_, 75 MHz] δ 167.5, 164.6, 159.4, 151.6, 145.5, 141.5, 141.4, 136.7, 136.6, 130.8, 130.7, 128.9, 128.8, 128.5, 127.5, 118.9, 113.7, 111.6, 111.5, 86.7, 86.6, 68.8, 68.7, 62.9, 58.6, 58.4, 55.5, 55.1, 50.7, 43.4, 43.3, 24.8, 24.7, 24.6, 24.4, 24.3, 20.5, 20.4, 19.8, 12.3. ^31^P-NMR [CDCl_3_, 162 MHz] δ 149, 147.9. HRMS (ES^+^) C_41_H_52_N_5_O_8_P calculated: 773.3557; found: [M+Na]^+^ 796.8645.

### 3.4. RNA Synthesis

RNA strands containing no modifications and a **T**^L^ unit at position 2 were synthesized on the 0.2 µmol scale using LV200 polystyrene supports. 3′- **T**^L^-**T**^L^-modified RNAs were synthesized on the 1 μmol scale using CPG functionalized with **T**^L^ units as solid supports. All oligonucleotides were synthesized on an Applied Biosystems 394 synthesizer (Foster City, CA, USA) using commercially available reagents and 2′-*O*-TBDMS-5′-*O*-DMT-protected phosphoramidites (A^Bz^, G^dmf^, C^Ac^ and U). The coupling time was 15 min and the coupling yields of natural and modified phosphoramidites were >97% in DMT-ON mode. Phosphorothioate oligonucleotides (**ASP** and **SSP**) (Table 1) were purchased from Sigma-Aldrich. SiRNAs previously described by Terrazas *et al*. [44] and Vaish and colleagues [45] were used to design siRNA duplexes against *Renilla* gene and *ApoB* gene respectively.

### 3.5. Deprotection and Purification of Unmodified and Modified RNA Oligonucleotide

Every solid support was treated at 55 °C for 1 h with 1.5 mL of NH_3_ solution (33%) and 0.5 mL of ethanol. Then, the suspension was cooled to room temperature; the supernatant was transferred into a clean tube and subsequently evaporated to dryness using a Speedvac concentrator. The obtained residue was dissolved in 1 M TBAF in THF (85 µL per 0.2 µmol resin, 330 µL per 1 µmol resin) and incubated for 15 h at room temperature. Finally, 1 M triethylammonium acetate (TEEA) and water were added to the solution (0.2 µmol synthesis: 85 µL of 1 M triethylammonium acetate (TEEA) and 330 µL water; 1 µmol synthesis: 330 µL 1 M TEEA and 830 µL water). Oligonucleotide desalting procedure was conducted on NAP-5 (0.2 µmol synthesis) or NAP-10 (1 µmol synthesis) columns using water as eluent and evaporated to dryness. The purification of oligonucleotides was carried out by HPLC (DMT-ON). Column: Nucleosil 120–10 C18 column (250 × 4 mm). Solvent A: 5% ACN in 0.1 M aqueous TEAAc (pH = 7) and solvent B: 70% ACN in 0.1 M aqueous TEAA (pH = 7). Flow rate: 3 mL/min. Conditions: 20 min linear gradient from 15% to 80% B and 5 min 80% B. The collected pure fractions were evaporated to dryness and then treated with 1 mL of 80% AcOH solution and incubated at room temperature for 30 min. The deprotected oligonucleotides were desalted on NAP-10 column using water as eluent. The resulting oligonucleotides were quantified by absorption at 260 nm and confirmed by MALDI mass spectrometry. SiRNA duplexes were prepared by annealing equimolar ratios of the sense and the antisense strands in siRNA suspension buffer (100 mM KOAc, 30 mM HEPES-KOH, 2 mM MgCl_2_, pH 7.4) at final concentration of 20 μM. Duplexes were heated at 95 °C for 5 min and slowly cooled to 4 °C.

### 3.6. Thermal Denaturation Studies

Melting curves of duplex RNA were performed following change of absorbance at 260 nm *versus* temperature. Samples were heated from 20 °C to 80 °C, with a linear temperature ramp of 0.5 °C/min in a V-650 spectrophotometer (JASCO, Easton, MD, USA) equipped with a Peltier temperature control. All the measurements were repeated thrice, both the heating and cooling curves were measured. Buffer condition: 100 mM KOAc, 30 mM HEPES-KOH, 2 mM MgCl_2_, pH 7.4.

### 3.7. Evaluation of Stability of RNAs to Exonucleases

3′-Exonuclease. Unmodified (**AS1**) and 3′-end modified (**AS2**) ssRNA (120 pmol) were incubated with Phosphodiesterase I (SVPD) from *Crotalus adamanteus* venom (EC 3.1.4.1) at 37 °C, as previously described [44]. Briefly, at established times, aliquots of the reaction mixtures (5 µL) were added to a solution of 9 M urea (15 µL) and immediately frozen at −80 °C. Samples were run on a denaturing 20% polyacrylamide TBE gel containing urea (7 M) and visualized by Sybr Green I staining.

5′-Exonuclease. Unmodified (**SS1**) and position 2 modified (**SS4**) ssRNAs (300 pmol) were incubated with Bovine Spleen Phosphodiesterase II (BSP) (EC 3.1.16.1) (0.37 U), in a solution containing NaOAc 100 mM (pH6.5) at 37 °C (total volume 50 µL). At certain times, aliquots of the reaction mixtures (5 µL) were added to a solution of 9 M urea (15 µL) and immediately frozen at −80 °C. Samples were run on a denaturing 20% polyacrylamide TBE gel containing urea (7 M) and visualized by Sybr Green I staining (Sigma Aldrich).

### 3.8. Serum Nucleases Stability Assay

Ds-RNAs containing no modifications, two **T**^L^ units and two phosphorothioate linkages at both 3′-ends (**AE1**, **AE4** and **AES4** respectively; 150 pmol) were incubated with 90% human serum at 37 °C. At appropriated times, aliquots of the reaction mixtures were separated, added to a glycerol loading solution and immediately frozen at −80 °C. Then, all samples were run on a non-denaturing 20% polyacrylamide TBE gel and visualized by Sybr green I staining. As dsRNA ladder, siRNA marker (New England Biolabs, Ipswich, MA, USA) was used.

### 3.9. Cells

HeLa cells (ATCC), HepG2 cells (ATCC), MEF^wt^ cells (ATCC) and MEF^Ago2−/−^ cell lines (a gift of Dr. O’Carroll [46]) were maintained in monolayer culture at exponential growth in high-glucose Dulbecco modified Eagle medium (DMEM) (Gibco, Life Technologies, Carlsbad, CA, USA) supplemented with 10% heat inactivated fetal bovine serum (Gibco, Life Technologies) and 1x penicillin/streptomycin solution (Gibco, Life Technologies), HeLa H/P cells stably expressing pGL4.14 [luc2/Hygro] (Promega) and pRL-tk-Puro (a kind gift of Dr. Waaler [47]) were maintained under hygromycin B (200 µg/mL) and puromycin (2 µg/mL) selection pressure. Human THP-1 monocyte cell line, a kind gift of Dr. Noé (University of Barcelona, Spain) and THP-1 iGLuc C1 cell line stably transduced with iGLuc reporter (pro-IL-1beta-GLuc-Flag) (kindly provided by Dr. Hornung [42]) were maintained in suspension culture at exponential growth with HAM F12 medium (Gibco, Life Technologies) supplemented with 10% heat inactivated fetal bovine serum and 1*×* penicillin/streptomycin solution. All cell lines were incubated at 37 °C in humidified environment with 5% CO_2_ and periodically checked for the presence of mycoplasma contamination. Cell viability was monitored by Trypan Blue exclusion assay and was higher than 95% in all experiments.

### 3.10. Luciferase Assay

For the luciferase assay, HeLa cells were plated in 24-well tissue culture plates at density of 1 × 10^5^ cells per well 24 h before transfection. Co-transfection of reported plasmids and different siRNAs molecules was performed using Lipofectamine 2000 (Life Technologies) in accordance with manufacturer’s instructions. Specifically, combinations of 1 µg of pGL3 (Promega, Madrid, Spain), containing the *Photinus pyralis* luciferase gene; 0.1 µg of pRL-TK (Promega), containing the *Renilla reniformis* luciferase gene and siRNAs at different concentrations siRNAs (1 nM, 0.3 nM, 0.16 nM, 60 pM, 16 pM, 8 pM, and 2 pM) were co-transfected. The siRNAs are designed to target the *Renilla* gene (accession number: M63501) in the 510–528 bp range. The inhibitory effects of siRNAs on *Renilla* protein expression was determined on lysates collected 24 h after transfection using the Dual-Luciferase Reporter Assay System (Promega) and a SpectraMax M5 luminometer (Molecular Devices, Sunnyvale, CA, USA). The ratios of *Renilla* luciferase (hRluc) to *Photinus* luciferase (luc+) protein activities were normalized to mock transfection, as control, and the mock activity was set as 100%. The results are representative of at least three independent experiments and each transfection was performed in triplicate. IC_50_ values were calculated by using GraphPad Prism software with the sigmoidal dose-response function.

### 3.11. Ago2-Mediated Silencing Assay

MEF^wt^ and MEF^Ago2−/−^ cells were plated in 24-well tissue culture plates at a density of 0.8 × 10^5^ cells per well 24 h before transfection. Co-transfection of reported plasmids (1 µg of pGL3 and 0.1 µg of pRL-TK) and different siRNAs molecules (**AE1** and **AE4**) at concentration of 1 nM and 16 pM was performed with lipofectamine LTX (Life Technologies) in accordance with manufacturer’s protocol for MEF. After 24 h, the samples were harvested for RNA extraction.

### 3.12. MTT Assay

The MTT assay is based on the protocol previously described [48]. Briefly, HeLa cells were seeded at density of 6 × 10^3^ per well into 96-well plate. After 24 h, naked siRNAs (**AE1**, **AE4** and **AES4**) and siRNAs complexed with lipofectamine 2000 (**AE1L**, **AE4L** and **AES4L**) were added to cells at different concentrations (1 nM, 10 nM, 50 nM, 100 nM). The culture medium after 24 h of incubation was changed and cells were incubated for 4 h with 0.8 mg/mL of MTT reagent. Finally, after washing with PBS, 200 µL of DMSO were added, gentle shaking for 15 min permitted the complete dissolution of formazan crystals. Absorbance was recorded at λ = 570 nm using the microplate spectrophotometer system SpectraMax M5 (Molecular Devices). Results were analyzed with GraphPad Prism software and are presented as percentage of the control values.

### 3.13. THP-1 Interferon Assay

THP-1 cells were seeded into 24-well tissue culture plates at density of 2 × 10^5^ cells per well in culture medium without antibiotics. SiRNAs were diluted to the desired concentration (60 nM) in OptiMem serum free medium (Gibco, Life Technologies) and transfections were performed using Interferin (Poly Plus Transfection, Inc., New York, NY, USA) in accordance with manufacturer’s instructions. After 24 h, the samples were collected for subsequent RNA extraction. As positive control we used cells transfected with Poly (I:C) (50 ng/mL, Sigma-Aldrich), this dsRNA analogue is sufficient to activate both the inflammasome pathway and the Type I IFNs response [49].

### 3.14. Single-Stranded Antisense siRNA 5′-End Phosphorylation

Before transfection, 300 pmol of single stranded antisense siRNA (**AS1**; **AS2**; **AS3**; **AS5**; **AS6**) (ss-siRNA) were incubated for 90 min at 37 °C with 100 mM of ATP and T4 Polynucleotide kinase (3′-phosphatase minus) (New England Biolabs, Ipswich, MA, USA), then for 30 min at 65 °C to inactivate the enzyme, following the manufacturer’s instructions.

### 3.15. HepG2 Transfection

HepG2 cells were reverse-transfected in gelatin coated 6-well plates at density of 0.6 × 10^6^ cells per well with 60 nM of double-stranded or single-stranded antisense siRNA, targeting endogenous *ApoB* mRNA using Lipofectamine RNAiMAX Reagent (Life Technologies) and following the manufacturer’s instructions for HepG2 cells. For time course experiments, 24 h after transfection, cells were splitted and parallel cultures were maintained for 9 days without any further treatments. For interferon assays, cells were reverse-transfected as above with different concentrations of siRNAs (20, 60, 100 nM) and pellets were harvested after 24 h.

### 3.16. THP-1 iGluc C1

THP-1 iGluc C1 cells were seeded in 96-well plates (0.2 × 10^6^ cells per well) 4 h before transfection to permit cell attachment. Then, the cells were transfected with 20 nM of either double-stranded siRNAs (**APO1** and **APO6**) or single-stranded siRNAs (**AS5** and **AS6**) using Interferin reagent (Polyplus Transfection), following the manufacturer’s instructions for THP-1 cells. After 22 h the supernatants were collected and to measure the luminescence levels, equal volumes of supernatants and coelenterazine (4.4 µM, Life Technologies) were mixed, as previously described [43]. As positive control, cells were transfected with Poly (I:C) (50 ng/mL).

### 3.17. Isolation of RNA and RT-qPCR

According to the manufacturer’s protocols, total RNA was isolated from HepG2 and THP-1 cells using Gene Jet RNA (Thermo Fischer Scientific, Waltham, MA, USA) and from MEF^wt^ and MEF^Ago2−/−^ with TRIzol reagent (Invitrogen, Carlsbad, CA, USA). Then, extracted RNA was quantified by NanoDrop (Thermo Scientific). Of each RNA sample, 5 µg was treated with DNase I [DNase I (RNase free) New England Biolabs] following manufacturer’s instruction. Then, the reverse transcription reaction, 1 µg of total RNA, was carried out with random hexamer primers and Revertaid H minus RT enzyme (Thermo Scientific) according to the manufacturer’s instructions. First strand cDNA was subsequently diluted 4 times in nuclease-free water before addition of 1 µL of resulting cDNA to the real-time mixture. Real-time PCR was accomplished in a total volume of 20 µL, using Maxima SYBR Green protocol (Thermo Scientific) following to the manufacturer’s instructions. The reference gene GADPH was used as internal control. *Renilla* and *ApoB* silencing was calculated and represented as 2^−∆∆Ct^ method, where 2^−∆∆Ct^ = [(Ct gene of interest − Ct internal control) sample A − (Ct gene of interest − Ct internal control) sample B)]. *ISGs* induction was measured and represented as 2^−∆Ct^ method, where 2^−∆Ct^ = [(Ct gene of interest − Ct internal control)]. All primers listed in Table S1 were purchased from Sigma-Aldrich and Primer-Blast was used as primer designing tool [50]. Furthermore, to verify the specificity and the identity of the PCR products and to exclude the formation of primer-dimers, for each pair of primers, melting curve analyses were performed. As negative control, No-template controls (NTCs) were included. Thermal cycling conditions: 95 °C for 10 min, followed by 50 cycles of 95 °C for 15 s, 60 °C for 30 s, and 72 °C for 30 s, and concluded at 72 °C for 10 min.

### 3.18. Western Blot Analysis

Cells treated with ApoB siRNAs were harvested at certain times, washed in PBS and lysed with 100 µL of lysis buffer (50 mM Tris-HCl pH8, 150 mM NaCl, 1% (v/v) NP-40, 0.1% (v/v) SDS, 0.25% (v/v) DOC) at 4 °C for 1 h. Protein concentrations were determined using the Bio-Rad protein assay system (Bio-Rad laboratories, Madrid, Spain). Aliquots of cell extracts, containing 20 µg of proteins, were denatured at 95 °C for 5 min in Laemmli buffer and resolved by 5% SDS-Page, and then blotted on a poly(vinylidene difluoride) membrane (Immobilion-P, Millipore, Milford, MA, USA). Membranes were blocked for 1 h in 1× TBST buffer (20 mM Tris-HCl pH 7.4, 150 mM NaCl, 0.20% (v/v) Tween-20) containing 5% (w/v) skim milk and subsequently incubated with the mouse anti-APOB antibody (ab63960, Abcam, Cambridge, UK) at 4 °C overnight at dilution 1:2000 in blocking buffer. The loading control β-actin was detected by mouse anti-Beta-actin antibody (Sigma-Aldrich) and diluted in blocking buffer 1:3500. Blots were washed 3 times with 1× TBST buffer and then incubated with the HRP-conjugated secondary antibody (anti-mouse IgG, W2041, Promega) for 1 h at room temperature. After 3 times washing with 1× TBST buffer, blots were incubated with ECL Western Blotting Substrate (Pierce, Rockford, IL, USA). Chemiluminescence was quantified as the ratios of ApoB to Beta-actin signal intensities and values obtained from siRNA-treated HepG2 cells were normalized to those obtained from HepG2 cells incubated in absence of siRNA.

### 3.19. Statistical Analysis

Statistical analysis was performed using GraphPad Prism software (GraphPad, San Diego, CA, USA). Unless otherwise noted, datasets were analyzed for statistical significance by one-way ANOVA and two-way ANOVA to generate P-values. IC_50_ determination was performed using non-linear regression analysis (log [inhibitor] *vs.* normalized response). Finally, human serum half-lives were determined using two phase decay equation. Quantifications of western blots and degradation assays were accomplished using ImageJ 1.46 software (NIH, Bethesda, MD, USA). For 3′-exonuclease, 5′-exonuclease and human serum nucleases stability experiments, intact bands percentage values were measured as the ratios of time specific band to time zero band intensities.

## 4. Conclusions

In this study we have analyzed the effect of the presence of an acyclic DNA mimetic at the 3′-ends of siRNAs. As the 3′-overhangs positions are not base paired, thermal stability of duplex siRNAs is not affected by this modification (Table 2). The different IC_50_ values emerged from the dual luciferase experiments reveal that all siRNAs used in our studies (**AE1**, **AE2**, **AE3**, **AE4**, **AES2**, **AES3**, and **AES4**) are active and potent inhibitors of *Renilla* luciferase expression. Even though the **AE2** and **AE4** siRNAs retain stronger activity compared to native siRNA (**AE1**), were found to be as effective as the **AES2** and **AES4** (Figure 2A). But at longer time the **AE4** siRNA retained more persistence silence capability compared to the **AES4** (Figure 3). Typically, siRNAs carrying modifications on the antisense 3′-overhang show silencing activity comparable to or lower than that of siRNAs [44,51,52,53,54,55] and only a few reports have described an improved silencing activity [56]. Interestingly, the siRNA modified on the antisense strand (**AE2**) demonstrates a better activity than the siRNA modified on sense strand (**AE3**). The higher silencing activity of **AE2** and **AES2** could be due to a better recognition of the 2 **T**^L^-modified and 2 **PS**-modified on 3′-overhang by the PAZ domain of the Ago2 protein [51,55,57,58]. Then, the stronger potency of the modified ss-siRNA **AS2P** respect to the **AS1P** underlines the real involvement of the L-threoninol-thymine modification to greater RNAi activity and also confirms the compatibility of the modification with the RNAi machinery (Figure 2B). Furthermore, data gathered from MEF^wt^ and MEF^Ago2−/−^ knockdown experiments, corroborate the thesis that gene silencing induced by l-threoninol modified siRNA (**AE4**) depends, as the native siRNA (**AE1)**, through Ago2-mediated mechanism (Supplementary Figure S8). Finally, the absence of cytotoxicity of the l-threoninol modification evaluated by the MTT assay (Supplementary Figure S9), excludes that the silencing siRNA-mediated is due to unspecific effects such as the protein synthesis inhibition and makes this modification appropriate for *in vivo* and *in vitro* applications. The good levels of RNAi activity displayed by our **T**^L^-modified siRNAs prompted us to analyze the specific contribution of the **T**^L^ modification on protection against exo/endo-nucleases. Nuclease resistance is a main issue during the delivery of naked siRNAs, unsatisfactory serum stability implies impracticability of systemic therapeutic strategies. The human serum experiment restates the extremely short half-life of native siRNAs (**AE1**), and proves the considerable superior resistance conferred by the 2 **T**^L^ units at both 3′-ends (**AE4**) even against the RNA protected at both 3′-ends with two phosphorothioate linkages (**AES4**) (Figure 4B). Thereafter analyses of the 3′- and 5′-exo degradation pattern revealed a significant increase in stability to specific phosphodiesterases such as Snake venom phosphodiesterase I and Bovine spleen phosphodiesterase II. Moreover, the analysis of double (**APO1** and **APO6**) and single-stranded (**AS5** and **AS6**) siRNAs targeting the endogenous APOB gene provides an exhaustive overview on the **T**^L^ modification compatibility with the endogenous RNAi machinery and also proves the longer silencing duration achieved by the presence of the modification especially in ss-siRNAs fashion. Finally, the l-threoninol modified siRNAs (**APO6** and **AS6**) are less prone to activate the IL-1beta production than the unmodified siRNAs (**APO1** and **AS5**), thus the replacement of natural thymidine with l-threoninol-thymine monomer can attenuate the activation of the immune response. In conclusion, evidence on the long-lasting silencing, the enhanced nucleases resistance, the absence of cytotoxicity, the silencing Ago2-mediated and less immunogenicity of the l-threoninol modified siRNA, suggests that this modification is a good candidate for further investigations involving *in vivo* and structural studies.

## Supplementary Materials

Supplementary materials can be accessed at: http://www.mdpi.com/1420-3049/19/11/17872/s1.

## Supplementary Files

Supplementary File 1
