# Supplementary material for: RNA/aTNA Chimeras: RNAi Effects and Nucleases Resistance of Single and Double Stranded RNAs
Source: Molecules. 2014 Nov 4;19(11):17872–96. doi: 10.3390/molecules191117872 (PMC6271724; doi:10.3390/molecules191117872)
Supplement: Supplementary File 1 [file molecules-19-17872-s001.pdf]

Supplementary Materials (ESI)

NMR SPECTRA

Figure S1. ^{1}H-NMR spectrum of compound **2**.

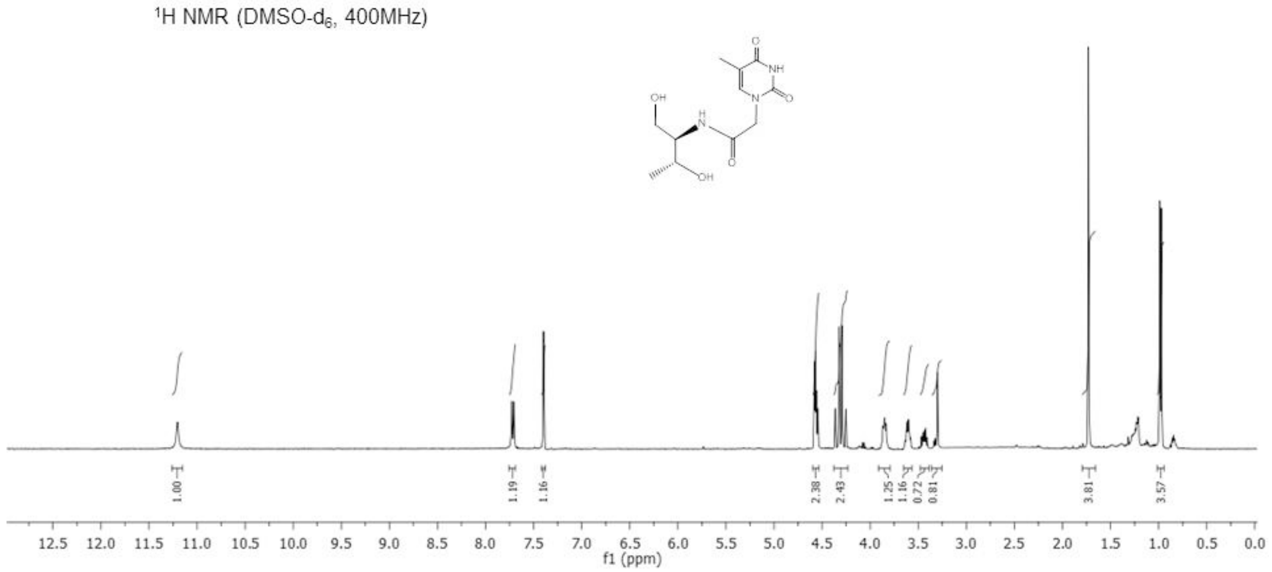

Figure S2. ^{1}H-NMR spectrum of compound **3**.

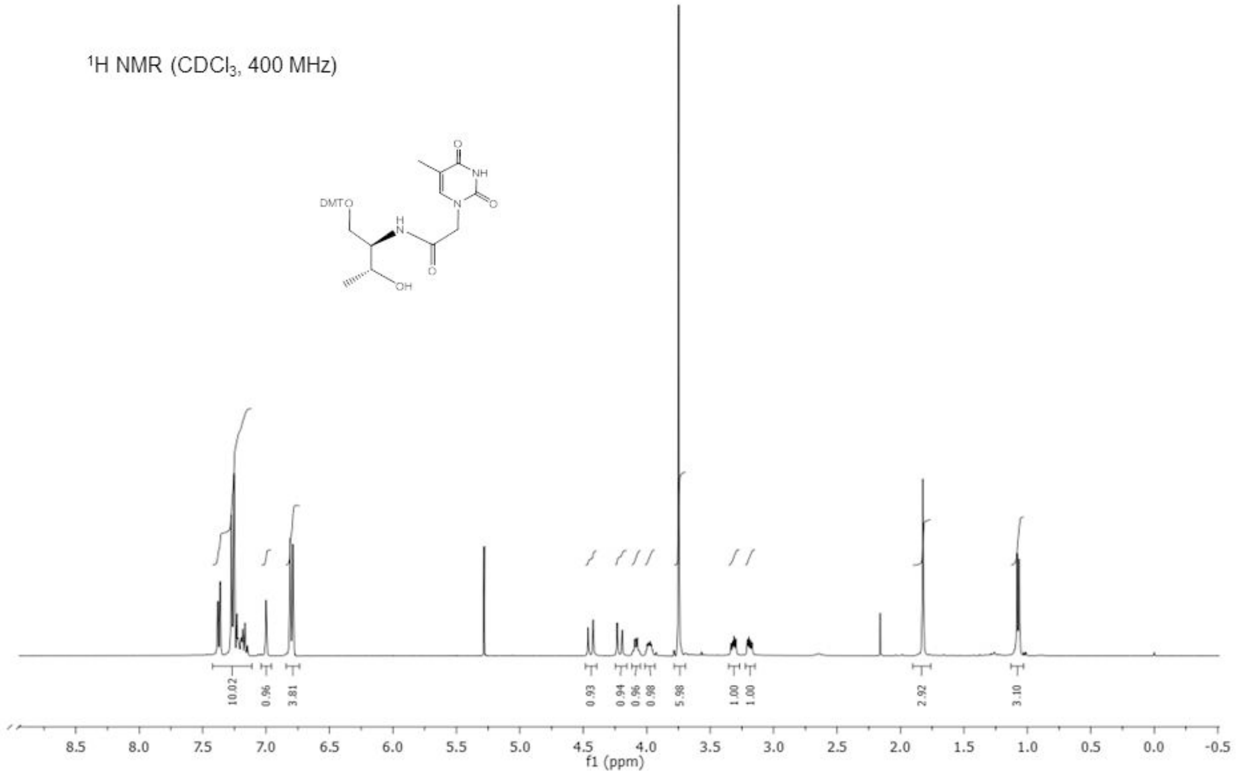

Figure S3. ^{13}C-NMR spectrum of compound **3**.

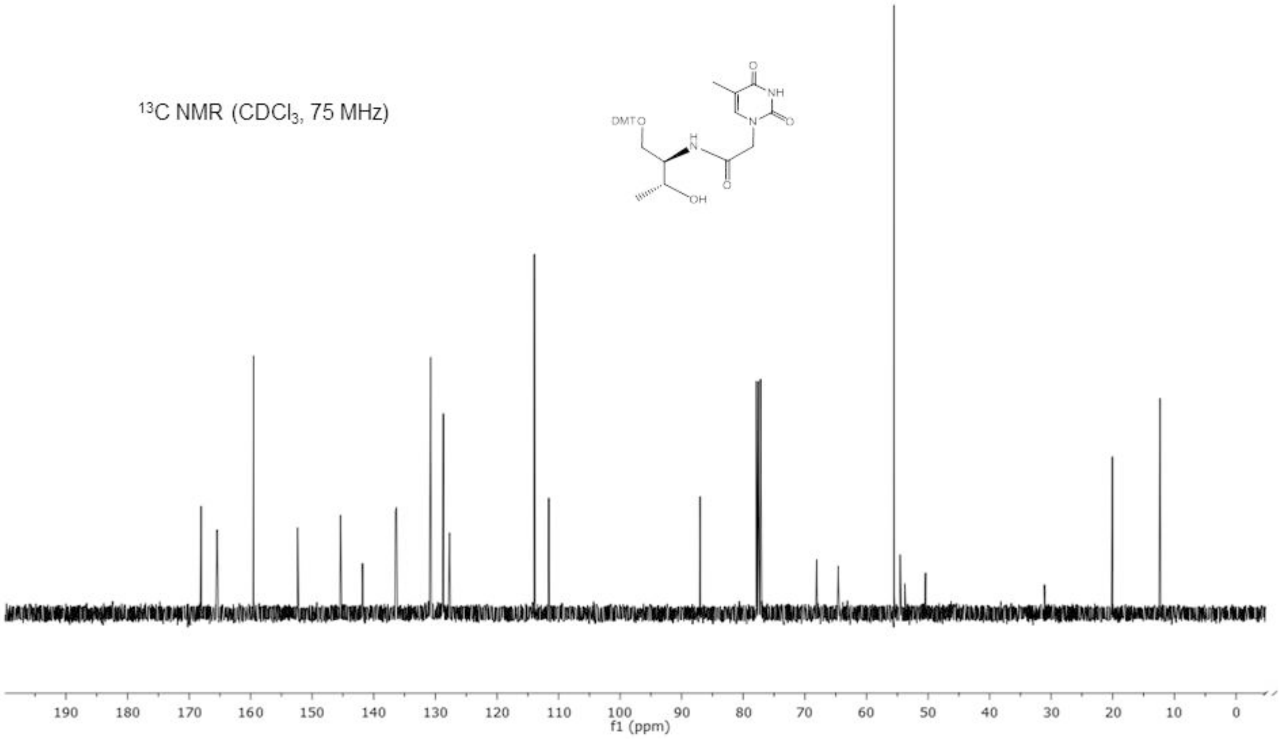

Figure S4. ^{1}H-NMR spectrum of compound **5**.

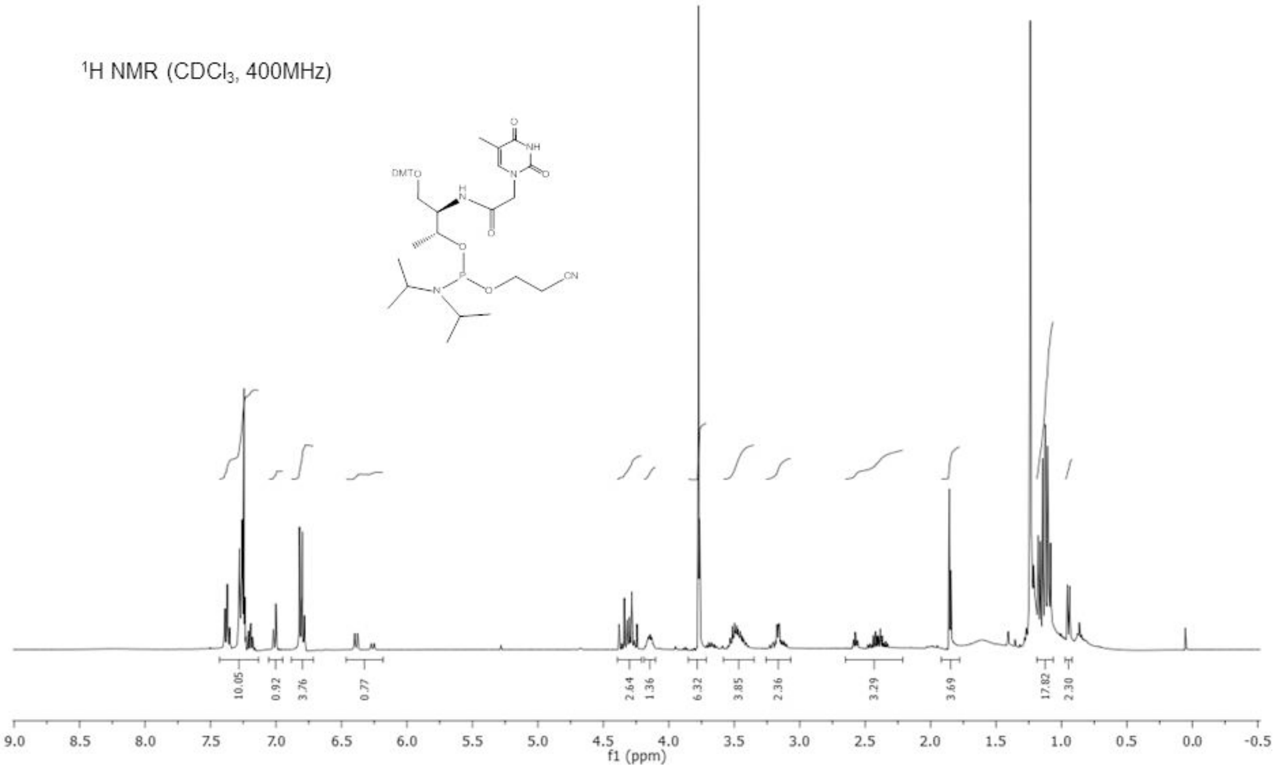

Figure S5. ^{13}C-NMR spectrum of compound **5**.

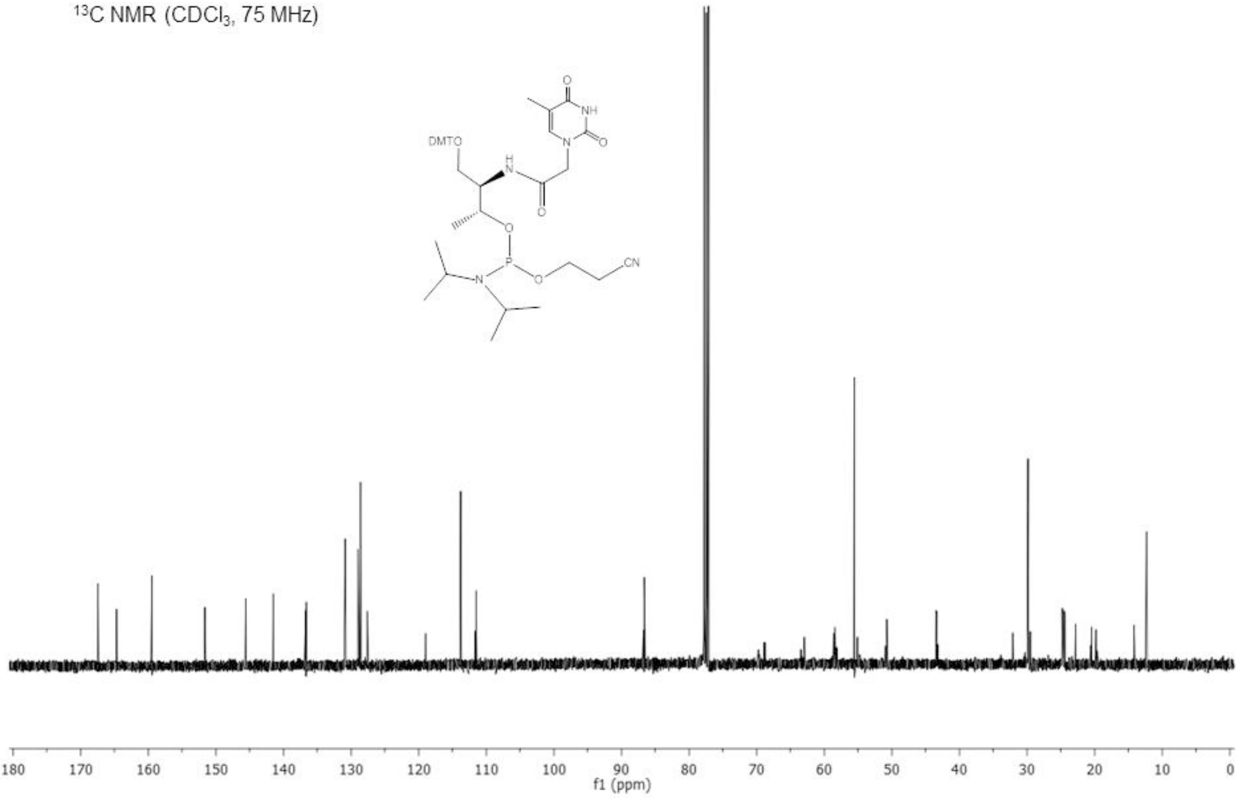

Figure S6. ^{31}P-NMR spectrum of compound **5**.

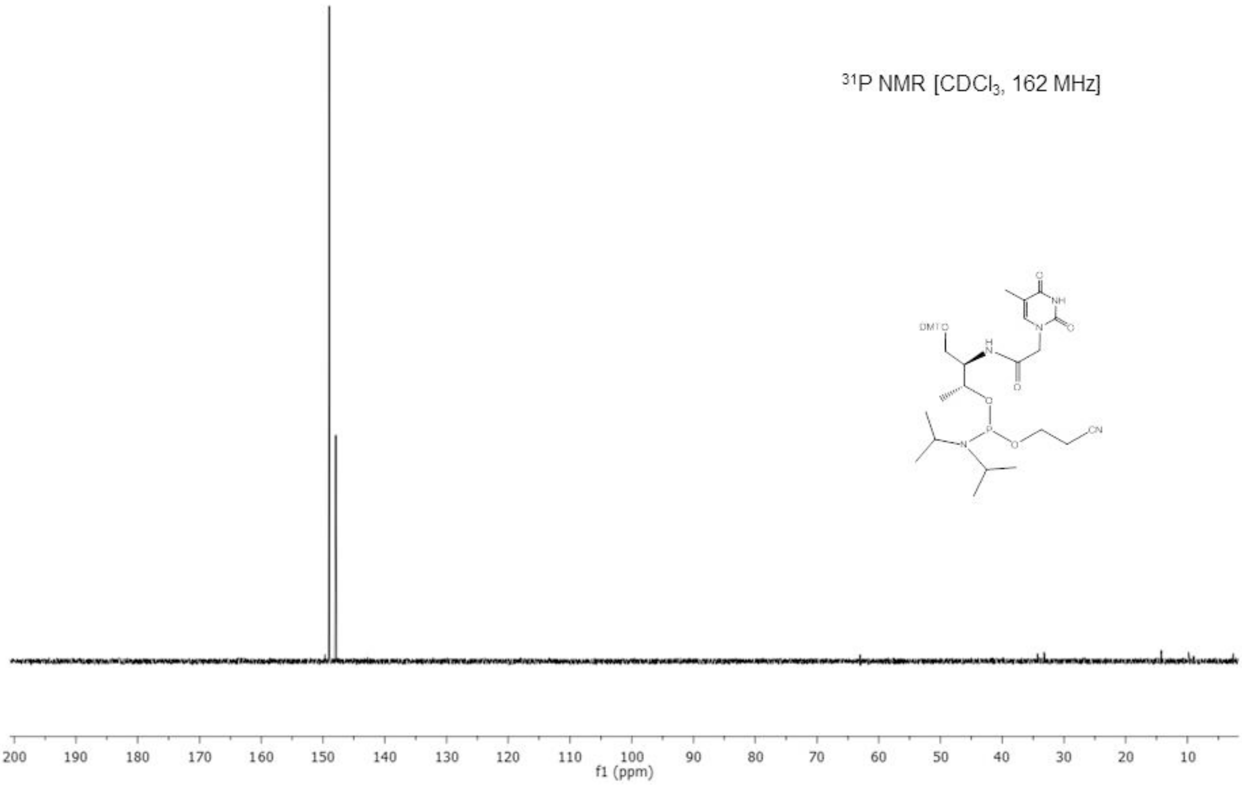

Figure S7. RNAi activity of non-phosphorylated ss-siRNAs. (**A**) Luminescence measurement 24 hours post-transfection of unmodified (**AS1**), modified (**AS2**) and scrambled (**AS3**) ss-siRNAs in HeLa cells. Values are mean ±SD, n = 3. (**B**) *ApoB* mRNA levels in HepG2 cells treated with 60 nM of unmodified (**AS5**) and modified (**AS6**) ss-siRNAs. Values are mean ±SD, n = 3.

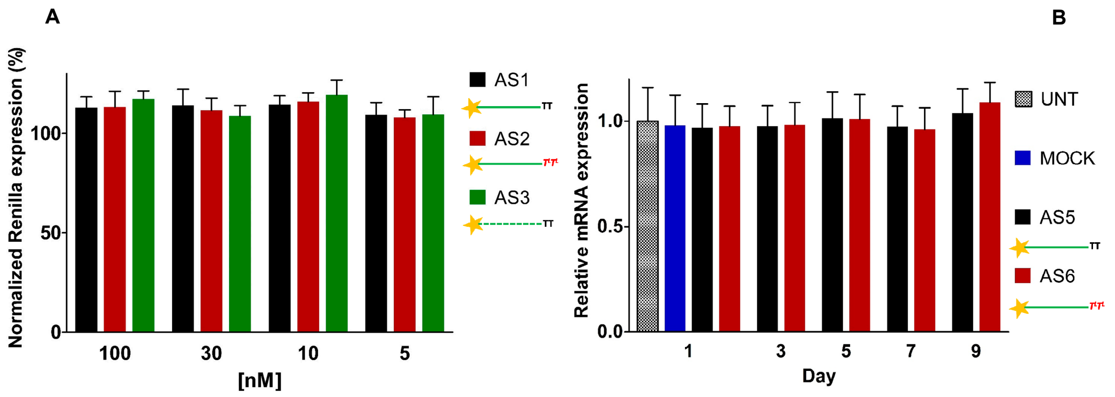

Figure S8. Silencing of *Renilla* gene Ago2-mediated. *Renilla* mRNA down-regulation in (**A**) MEF$^{Ago2-/-}$ and (**B**) MEF^{wt} cells transfected with 1 nM and 0.016 nM of unmodified **AE1** and modified **AE4**. n = 3 ±SD. For experimental conditions see Material and Methods.

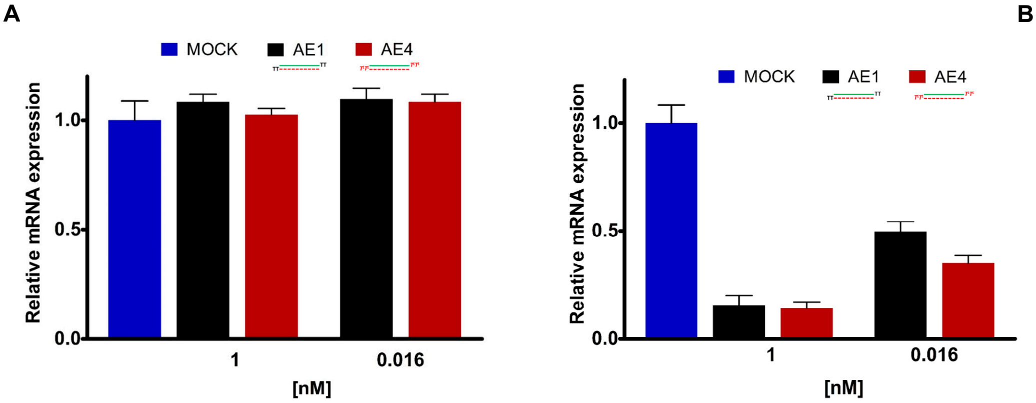

Figure S9. Cytotoxic effect of L-threoninol siRNA on HeLa cells survival. MTT assay showing cell proliferation 24 hours post-transfection with unmodified and modified siRNAs with or without transfection reagent (**AE1L**, **AE6L**, **AE1** and **AE6** respectively). Data are shown as mean ±SD, n = 5.

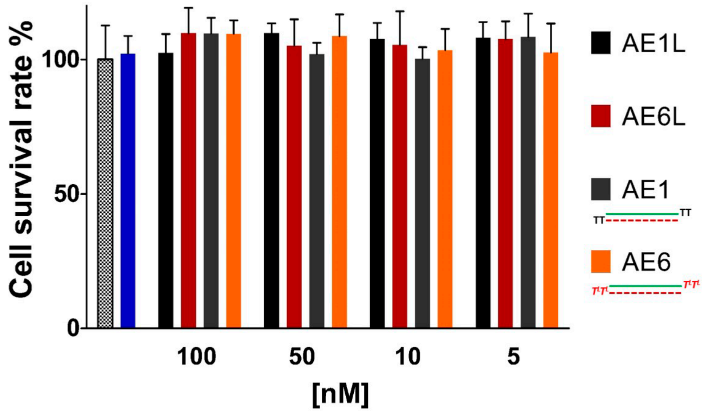

Figure S10. Native TBE gel of native siRNA (**AE1**). **Lane 1:** dsRNA ladder; **lane 2:** 300 pmol of **AE1** without human serum (**No HS**); **lane 3-9: AE1** degradation pattern over the time of 24 hours in presence of 90% of human serum.

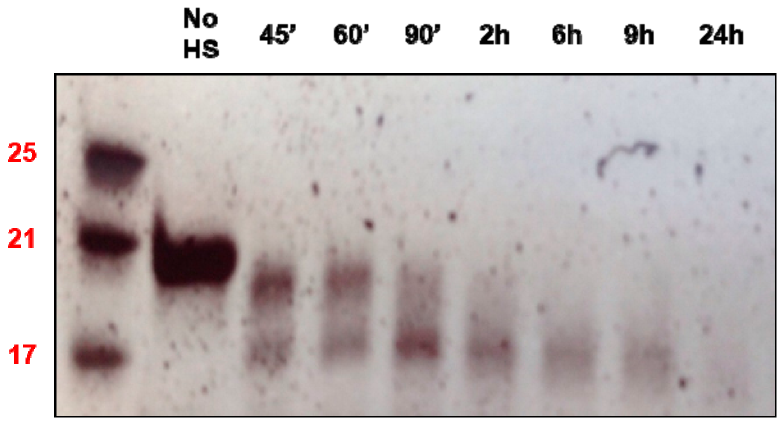

Figure S11. Quantitative RT-PCR analysis of selected *ISGs*: *ISG-56*, *PKR*, *IFITM1*, *MX1*, *OAS-1*. (**A**) mRNA levels after HepG2 transfection of unmodified (**APO1**) siRNA and double-modified (**APO6**) siRNA. (**B**) mRNA levels after THP-1 transfection of unmodified (**APO1**) siRNA and double-modified (**APO6**) siRNA. Results are presented as mean ±SD, n = 3. For experimental conditions see Material and Methods. *ns* = $p > 0.05$; * = $p < 0.05$; ** = $p < 0.01$; *** = $p < 0.001$.

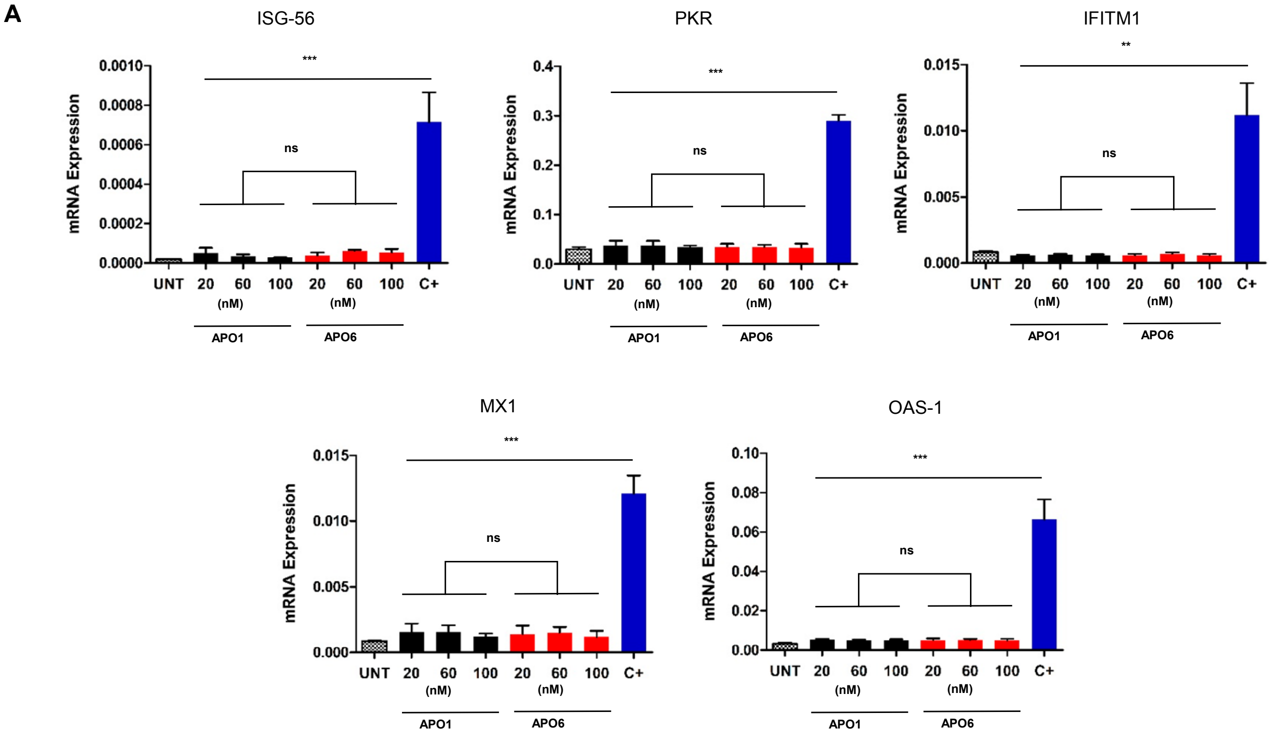

Figure S11. *Cont.*

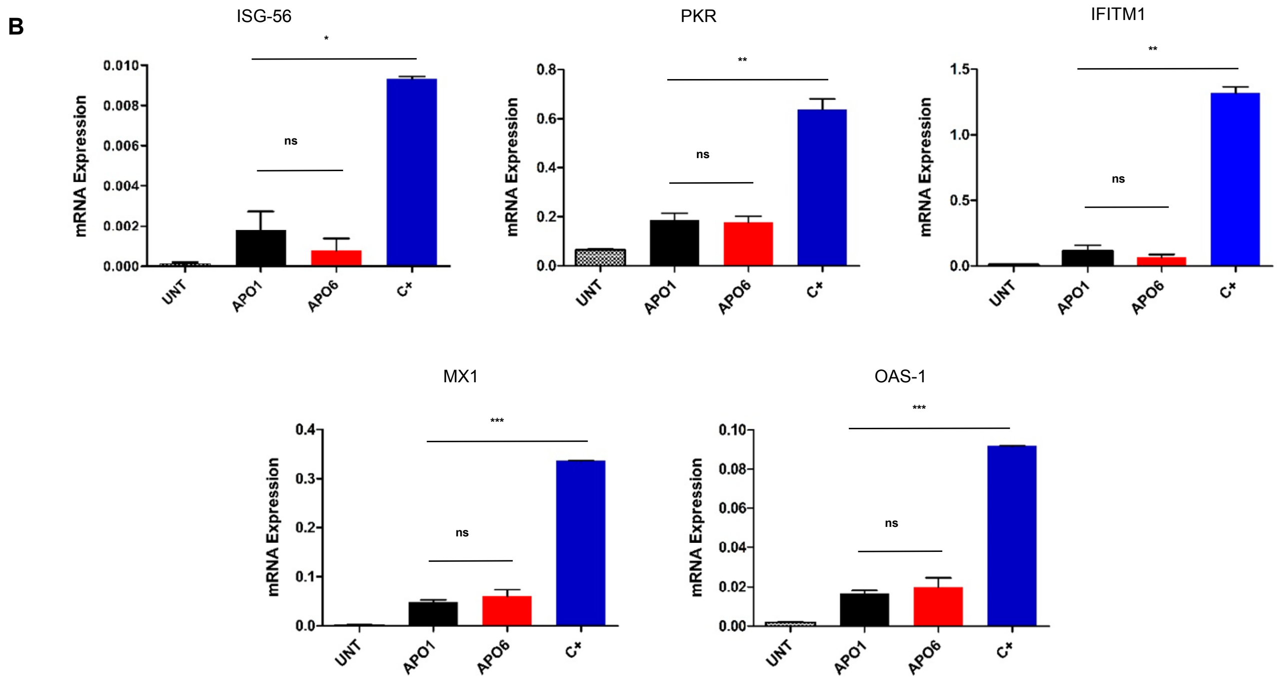

Table S1. List of primers.

	Forward	Reverse
**GAPDH**	5'-TGCACCACCAACTGCTTAG	5'-GATGCAGGGATGATGTTC
**APOB**	5'-CAAAGCCACCCTGGAACTCT	5'-CTGCAATGTCAAGGTGTGCC
**PKR**	5'-ACTTT TTCCTGGCTCATCTC	5'-ACATGCCTGTAATCCAGCTA
**ISG56**	5'-TTCGGAGAAAGGCATTAGA	5'-TCCAGGGCTTCATTCATAT
**MX1**	5'-CAGCACCTGATGGCCTATCA	5'-TGGAGCATGAAGAACTGGATGA
**OAS-1**	5'-AGAAGGCAGCTCACGAAACC	5'-CCACCACCCAAGTTTCCTGTA
**IFITM-1**	5'-GACAGGAAGATGGTTGGCGA	5'-GGTAGACTGTCACAGAGCCG
**RL-TK**	5'-GTGGTGGGCCAGATGTAAAC	5'-CAGGTGCATCTTCTTGCGAA
